# Mapping the tumor microenvironment in clear cell renal carcinoma by single-cell transcriptome analysis

**DOI:** 10.3389/fgene.2023.1207233

**Published:** 2023-07-18

**Authors:** Yuxiong Wang, Yishu Wang, Bin Liu, Xin Gao, Yunkuo Li, Faping Li, Honglan Zhou

**Affiliations:** ^1^ Department of Urology, The First Hospital of Jilin University, Jilin, China; ^2^ Key Laboratory of Pathobiology, Ministry of Education, Jilin University, Jilin, China

**Keywords:** intratumoral heterogeneity, clear cell renal cell carcinoma, copy number variation, single-cell RNA sequencing, cancer-associated fibroblasts, tumor-associated macrophages

## Abstract

**Introduction:** Clear cell renal cell carcinoma (ccRCC) is associated with unfavorable clinical outcomes. To identify viable therapeutic targets, a comprehensive understanding of intratumoral heterogeneity is crucial. In this study, we conducted bioinformatic analysis to scrutinize single-cell RNA sequencing data of ccRCC tumor and para-tumor samples, aiming to elucidate the intratumoral heterogeneity in the ccRCC tumor microenvironment (TME).

**Methods:** A total of 51,780 single cells from seven ccRCC tumors and five para-tumor samples were identified and grouped into 11 cell lineages using bioinformatic analysis. These lineages included tumor cells, myeloid cells, T-cells, fibroblasts, and endothelial cells, indicating a high degree of heterogeneity in the TME. Copy number variation (CNV) analysis was performed to compare CNV frequencies between tumor and normal cells. The myeloid cell population was further re-clustered into three major subgroups: monocytes, macrophages, and dendritic cells. Differential expression analysis, gene ontology, and gene set enrichment analysis were employed to assess inter-cluster and intra-cluster functional heterogeneity within the ccRCC TME.

**Results:** Our findings revealed that immune cells in the TME predominantly adopted an inflammatory suppression state, promoting tumor cell growth and immune evasion. Additionally, tumor cells exhibited higher CNV frequencies compared to normal cells. The myeloid cell subgroups demonstrated distinct functional properties, with monocytes, macrophages, and dendritic cells displaying diverse roles in the TME. Certain immune cells exhibited pro-tumor and immunosuppressive effects, while others demonstrated antitumor and immunostimulatory properties.

**Conclusion:** This study contributes to the understanding of intratumoral heterogeneity in the ccRCC TME and provides potential therapeutic targets for ccRCC treatment. The findings emphasize the importance of considering the diverse functional roles of immune cells in the TME for effective therapeutic interventions.

## 1 Introduction

Over the past two decades, there has been a steady annual increase of 2% in the global incidence of renal cell carcinoma (RCC), resulting in estimated new RCC cases exceeding 77,000 in China and 71,000 in the United States, with RCC-related deaths surpassing 46,000 and 15,000 in the respective countries in 2022 (Xia et al., 2022). Clear cell renal cell carcinoma (ccRCC) represents the predominant clinicopathological subtype, accounting for approximately 70%–80% of all RCC cases (Bray et al., 2018; Ljungberg et al., 2022). Despite advancements in tumor biology and therapeutic modalities, the long-term clinical prognosis of patients with ccRCC remains poor.

Rossi et al. (2022) have reported that tumor metabolic heterogeneity plays a key role in tumor invasion and metastasis and vascular endothelial cells (ECs) are involved in regulating tumor cell metabolic status. In addition to intrinsic factors of tumor cells, the interaction between tumor cells and other cell types within the tumor microenvironment (TME) contribute to tumor metabolic heterogeneity, influencing disease progression. For instance, single-cell sequencing indicated substantial variations in energy metabolism and oxidative phosphorylation among different clusters of tumor cells and intratumoral ECs in our bioinformatics study. Additionally, single-cell sequencing analysis revealed that immune cells, constituting approximately 30% of the total cells in ccRCC samples, exhibited diverse functional profiles, including tumor-promoting and anti-inflammatory effects (Zhang et al., 2021a). Although cytotoxic CD8^+^ T cells are a critical component of antitumor immune response, tumor cells can induce CD8^+^ T cell dysfunction through complex intercellular mechanisms, contributing to tumor immune escape in patients with ccRCC (Iwai et al., 2002; Wu et al., 2020; Borcherding et al., 2021). Cell function heterogeneity has also been observed and further studied in tumor-infiltrating immune cells. There are two types of tumor-associated macrophages (TAMs) in the TME: M1 and M2 (Mortezaee and Majidpoor, 2022). Analysis of single-cell sequencing data of clinical samples obtained from a publicly available transcriptome database revealed that TAMs displayed elevated expression levels of the immune checkpoint genes, namely, CD274 and CD276, which bind to receptors on the surface of T lymphocytes, consequently impairing their tumor-killing capacity. Moreover, the abundance of M2-like TAMs in the TME is significantly associated with adverse clinical outcomes (Hu et al., 2020). Notably, these complex M2-like macrophages were found to exhibit high cytokines, such as CCL3 and CXCL2, and the angiogenic factor VEGFA, indicating a paradoxical population of immunosuppressive and angiogenic macrophages in tumors with the ability to both inhibit adaptive immune responses and recruit immune cells (Young et al., 2018; Borcherding et al., 2021; Obradovic et al., 2021).

Although ccRCC is an immunogenic tumor, the underlying immunocytodynamics governing both antitumor and pro-tumor responses are not fully understood. High-throughput single-cell RNA sequencing (scRNA-seq) is a valuable tool for classifying various cell subpopulations in the TME, identifying representative gene expression signatures at the individual cell level, and describing the transcriptional status of different cell types. Compared to conventional bulk RNA sequencing, scRNA-seq has the potential to unveil the contributions of various cell populations within tumors and reveal the underlying mechanisms influencing tumor cell viability and progression (Obradovic et al., 2021). Tumor stromal cells, including tumor-infiltrating immune cells, ECs, and fibroblasts, have been reported to exhibit pronounced heterogeneity, which has been implicated in the limited response to targeted therapies among patients with malignancies (Papalexi and Satija, 2018). Therefore, a comprehensive understanding of the intratumoral landscape is necessary for effective treatment.

Here, we analyzed scRNA-seq data of 12 samples, including seven ccRCC tumor and five para-tumor samples to elucidate the intricate intratumoral heterogeneity prevalent in ccRCC. It is anticipated that these findings of this study will significantly contribute to the understanding of the biological characteristics of ccRCC, thereby laying the foundation for the implementation of individualized and precise treatment approaches tailored to ccRCC patients.

## 2 Materials and methods

### 2.1 Data correction and quality control

Raw scRNA-seq profiling dataset GSE156632 was downloaded from the GEO database (https://www.ncbi.nlm.nih.gov/geo/). A comprehensive set of 12 samples, including seven tumor and five para-tumor samples, was included in the analysis. Quality control measures were performed using the Seurat package (version 3.0.6) (Butler et al., 2018). Single cells characterized by mitochondrial gene content exceeding 10% or possessing fewer than 200 genes were excluded from further analysis. The harmony algorithm was applied to eliminate batch effect between the different samples. Finally, 51,780 single cells, comprising 18,682 cells derived from normal tissue and 33,098 cells derived from tumor tissue, were retained for further investigation. Additionally, we utilized two additional scRNA-seq profiles from Su et al. (2021) and Young et al. (2018), available in their Supplementary material, to validate certain findings. These profiles collectively encompassed 42,958 cells. Bulk RNA-seq data of ccRCC samples, including 533 tumor samples, were obtained from The Cancer Genome Atlas (TCGA) database (https://portal.gdc.cancer.gov/).

### 2.2 Dimensionality reduction and cell clustering

Dimensionality reduction and cell clustering analysis were performed on the sequencing data, and each cluster was visualized using 2D uniform manifold approximation and projection (UMAP). The main cell types were identified using markers obtained from the CellMarker database and previous studies (Lake et al., 2019; Zhang et al., 2019; Hu et al., 2020), and marker genes were visualized using dot plots or violin plots. The 51,780 cells were clustered into 11 major cell types, and each cell type was further clustered into subclusters to detect intracellular heterogeneity. Preferentially expressed genes in clusters or differentially expressed genes (DEGs) between tumor- and normal-derived cells were identified using the FindAllMarkers function in Seurat.

### 2.3 Estimation of copy number variations (CNVs) in epithelial cells

Estimation of CNVs in epithelial cells entailed employing the default parameters of the InferCNV package, with four clusters containing non-malignant derived proximal tubule epithelial cells as control. Heatmap illustrating the top 10 DEGs in each group was generated. Kaplan-Meier analysis of MALAT1 was performed using the online tool GEPIA to evaluate the prognostic value of highly expressed genes and detect the role of these genes in ccRCC progression.

### 2.4 Functional enrichment analysis

DEGs among cell clusters were identified using the FindAllMarkers function in Seurat, with cut-off threshold values of |log_2_(Fold Change)| > 0.25 and adjusted *p* < 0.05. Kyoto Encyclopedia of Genes and Genomes (KEGG) and gene ontology (GO) enrichment analyses of DEGs were performed using the DAVID (version 6.8) online tool (https://david.ncifcrf.gov/). Gene set variation analysis (GSVA) was conducted with the GSVA package. Metabolic gene sets obtained from a previously published study (Gaude and Frezza, 2016) was tested using the R limma package. Pathways with adjusted *p* < 0.05 were considered significantly enriched.

### 2.5 Statistical analysis

A paired *t*-test was performed to determine differences in the expression of CD4^+^ or CD8^+^ lymphocytes between tumor tissues and paired para-tumor tissues. Data were considered statistically significant at *p* < 0.05.

## 3 Results

### 3.1 Single-cell sequencing and cell typing of ccRCC and paired para-tumor tissues

**FIGURE 1:**
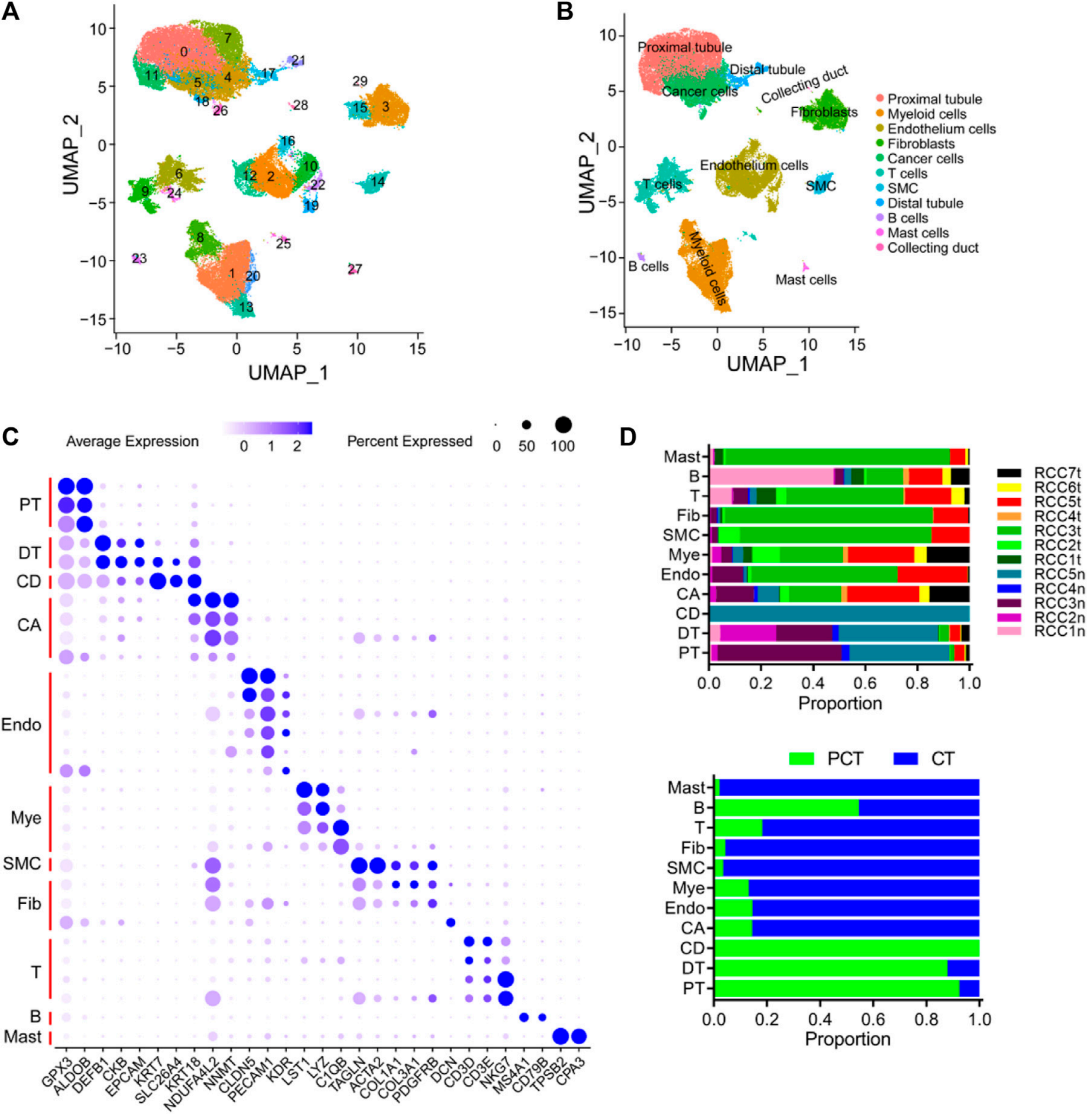
Overview of single cells derived from ccRCC and para-tumor samples. **(A, B)** UMAP plot of all the single cells derived from ccRCC and para-tumor samples, with each color coded for **(A)** 30 clusters of cells, and **(B)** 11 major cell types. **(C)** The cell-marker genes of 11 major cell types identified in this study. **(D)** The 11 cell types identified in this study (from top to bottom): The fraction of cells that originated from the five para-tumor and seven tumor samples, and the fraction of cells from sample origin type.

A cohort of 12 samples, encompassing seven renal cancer tissues and five paired para-tumor tissues, was collected from seven patients who underwent radical nephrectomy as part of this study. Rigorous quality control measures were implemented, including the removal of batch effects (Supplementary Figure S1). Subsequently, a total of 51,780 cells were identified including 18,682 normal tissue-derived cells and 33,098 tumor (ccRCC)-derived cells. A total of 20,531 genes from scRNA-seq validation data were analyzed. The 51,780 cells were classified into 30 clusters according to known cluster-specific genes described in previous literature, and the 30 clusters were typed into 11 cell lineages based on cell type-specific genes (Lambrechts et al., 2018; Chen et al., 2020; Hu et al., 2020). The identified cell lineages included proximal renal tubular cells (GPX3 and ALDOB), distal renal tubular cells (DEFB1, CKB, and EPCAM), collecting duct cells (KRT7, SLC26A4, and KRT18), cancer cells (NDUFA4L2 and NNMT), ECs (CLDN5, PECAM1, and KDR), myeloid cell (LST1, LYZ, and C1QB), smooth muscle cells (TAGLN and ACTA2), fibroblasts (COL1A1, COL3A1, PDGFRB, and DCN), T-cells (CD3D, CD3E, and NKG7); B cells (MS4A1 and CD79B), and mast cells (TPSB2 and CPA3) (Figures 1A–C). The overall distribution of these cell lineages across different patients and tissues is shown in Figure 1D, and was consistent with previous findings in kidney diseases (Muto et al., 2021; Xu et al., 2022a; Schreibing and Kramann, 2022). Specifically, tumor tissues exhibited a higher proportion of inflammatory cells, ECs, and fibroblasts compared to para-tumor tissues, indicating inter-tumor heterogeneity in the composition of stromal cells. Cancer cells, myeloid cells, T cells, fibroblasts, and ECs were re-clustered to analyze their roles in the occurrence and development of ccRCC. B cells, mast cells, and smooth muscle cells (SMC) were excluded from the re-clustering analysis due to their limited representation in the tumor samples, ensuring a more unbiased approach.

### 3.2 High CNV heterogeneity was observed in tumor cells

**FIGURE 2:**
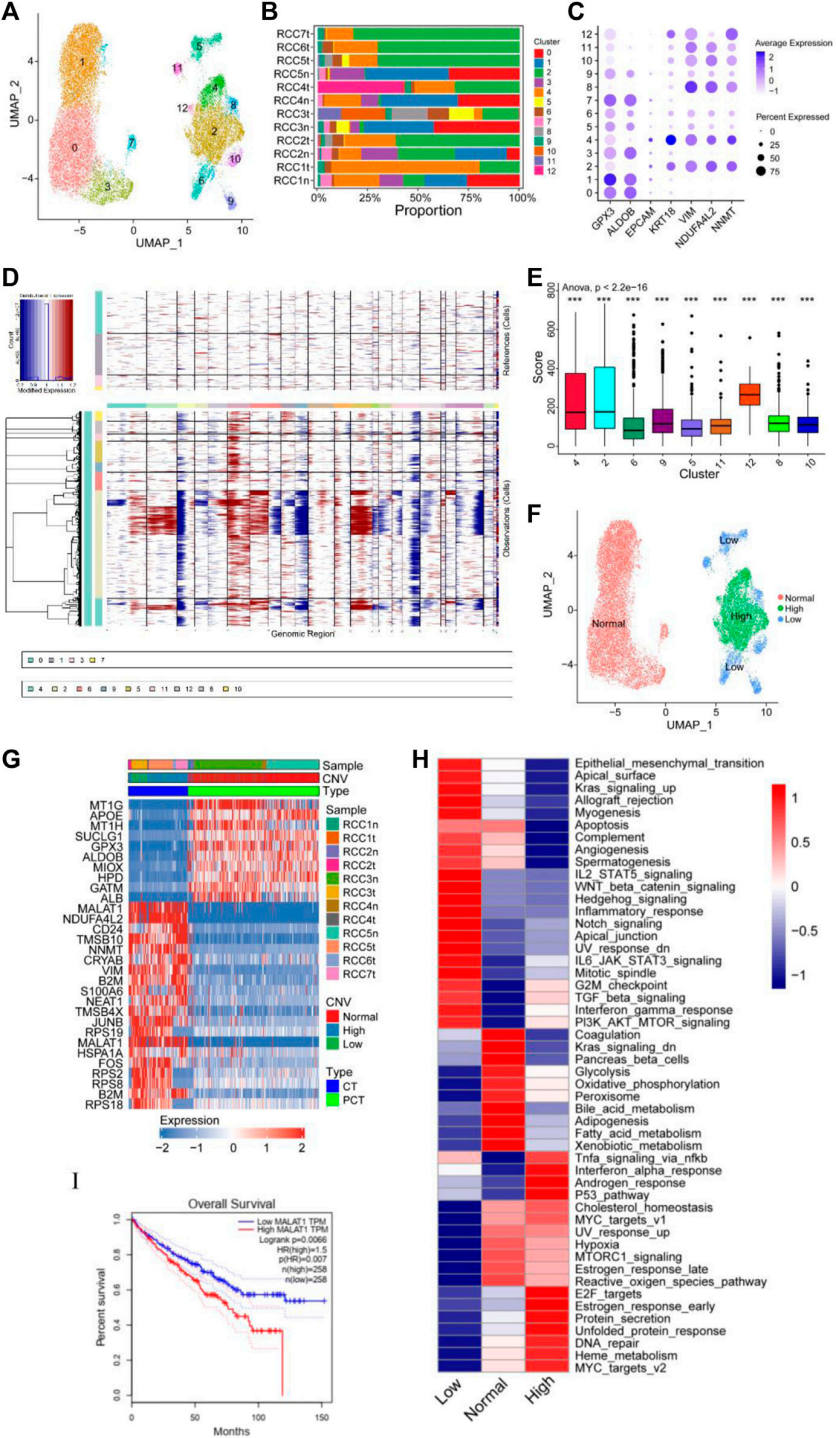
Copy number variation (CNV) heterogeneity was observed in tumor cells. **(A–C)** Re-clustering of tumor cells and proximal tubular cells. **(A)** UMAP plot of tumor cells and proximal tubular cells from Figure 1B. **(B)** For 13 cell clusters: fraction of cells that originated from the five para-tumor and seven tumor samples. **(C)** The marker genes for the 13 cell clusters. **(D–F)** Estimation of CNVs in cancer cells. **(D)** CNV analysis of each cluster of cells that originated from the five para-tumor (upper) and seven tumor samples (lower). **(E)** Box diagram of CNV score of each cluster of cells derived from the tumor samples. **(F)** UMAP plot of tumor cells and proximal tubular cells, colored according CNV level. **(G)** Heatmap of the top 10 differentially expressed genes (DEGs) between normal group, high CNV group, and low CNV group. **(H)** Differences in 50 hallmark pathway activities between the three groups were determined using GSVA package. Shown are t values calculated using a linear model. **(I)** Kaplan-Meier survival curve showing high level of MALAT1, which indicated poor prognosis in TCGA KIRC cohort. Log-rank *p* < 0.05 was considered as statistically significant.

The cancer and proximal tubular cells (Figure 1B) were re-clustered into 13 groups (Figure 2A), and the proportion of each cluster in the respective sample is depicted in Figure 2B. A total of 20,020 cells were reanalyzed, including 13,761 cells from para-tumor tissues and 6,259 cells from tumor tissues. This analysis was performed using specific marker genes: GPX3 and ALDOB for proximal tubular cells and VIM, KRT18, NDUFA4L2, and NNMT for tumor cells. Based on the expression of the annotated gene, clusters 0/1/3/7 were defined as normal tubular cells, while the remaining clusters were classified as tumor cells (Figures 2A, C). CNV analysis was performed using clusters 0/1/3/7 as the control group, and re-clustered cells were scored. Compared with normal tubular cells, there were more CNVs in tumor cells (Figures 2D, E). Moreover, the CNV scores exhibited considerable high heterogeneity within tumor cells. Therefore, the re-clustered cells were divided into three subgroups based on CNVs: high-CNV (cluster 2/4/12), low-CNV (cluster 5/6/8/9/10/11), and normal proximal tubular cell (control) groups. Consistent with the CNV score, the three groups of cells were classified in the UMAP plot (Figures 2E, F). Remarkably, amplifications of chromosomes 2, 7, and 12 and deletions of chromosomes X and 19 were detected in the high CNV group. Notably, deletion of chromosomes 3, 9, and 16 and amplification of chromosome 6 was observed in both the high and low CNV groups, indicating some level of CNV homogeneity in tumor cells (Figures 2E, F). The diverse metabolism and progression of ccRCC may be attributed to different chromosomal CNVs. Additionally, distinct CNVs were observed between tumor and non-tumor cells, further highlighting the significant CNV heterogeneity in tumor cells. Moreover, the CNV analysis of the scRNA-seq validation dataset confirmed the presence of CNV heterogeneity in tumor cells, as evidenced by amplification of multiple chromosomes (Borcherding et al., 2021; Borcherding et al., 2021; Obradovic et al., 2021; Ljungberg et al., 2022) and deletions of multiple chromosomes (Bray et al., 2018; Chen et al., 2020; Mortezaee and Majidpoor, 2022; Mortezaee and Majidpoor, 2022) (Supplementary Figure S2A).

Differential expression analysis was performed using the Limma package to identify DEGs within the three cell subgroups. A heatmap displaying the top 10 DEGs in each group was presented in Figure 2G. Significant variations in the expression levels of the top 10 genes were observed between normal tubular cells and tumor cells, whereas such differences were not statistically significant between the high and low CNV groups. Notably, MALAT1 emerged as one of the top 10 DEGs in both the high and low CNV groups. In ccRCC, MALAT1 has been implicated in various metabolic processes. It has been shown to interact with SCD, an enzyme involved in fatty acid biosynthesis, to participate in the regulation of lipid metabolism (Zhou et al., 2022) and lipid uptake and insulin resistance through multiple pathways (Yan et al., 2016; Zhao et al., 2021). In the TCGA-KIRC cohort, compared to normal tissues, tumor tissues exhibit higher levels of MALAT1 (Supplementary Figure S2B), and elevated MALAT1 expression was significantly positively associated with poor prognosis (Figure 2I).

GSVA revealed notable enrichments in specific biological pathways across different CNV groups. The high CNV group exhibited significant enrichment in Myc targets, DNA repair, mTOR signaling, and E2F targets compared to the control group. Conversely, the low CNV group showed pronounced enrichment in epithelial mesenchymal transition (EMT), Wnt/β-catenin signaling, and Notch signaling compared to the control group (Figure 2H). Moreover, TGF-β signaling, PI3K/AKT/mTOR signaling, G2/M checkpoint, and interferon gamma response were enriched in both CNV groups.

Tumor cells and control cells underwent re-annotated using cell cycle-related genes, and the results were visualized in a UMAP plot (Supplementary Figures S2A–C). Tumor cells predominantly occupied the G2/M phase, indicating a high level of cell proliferation, which was consistent with the enrichment of E2F targets and G2/M checkpoint. These findings supported the notion that ccRCC was characterized by distinct metabolic alterations, as evidenced by the enrichment of multiple enriched metabolic pathways (Figure 2H). Therefore, a detailed analysis of the metabolic pathways in the three groups was performed. Compared with the control group, lipid anabolism, drug metabolism, O-glycan synthesis, and N-glycan synthesis pathways were significantly enriched in the high and low CNV groups. In contrast, oxidative phosphorylation and the tricarboxylic acid cycle had low enrichment levels in the CNV groups (Supplementary Figure S3). These observations shed light on the metabolic landscape of ccRCC and highlight the distinctive metabolic features associated with chromosomal copy number variations.

### 3.3 Re-clustered myeloid cells had a negative immunoregulatory function in ccRCC

#### 3.3.1 Myeloid cells were classified into three cell types for analysis

**FIGURE 3:**
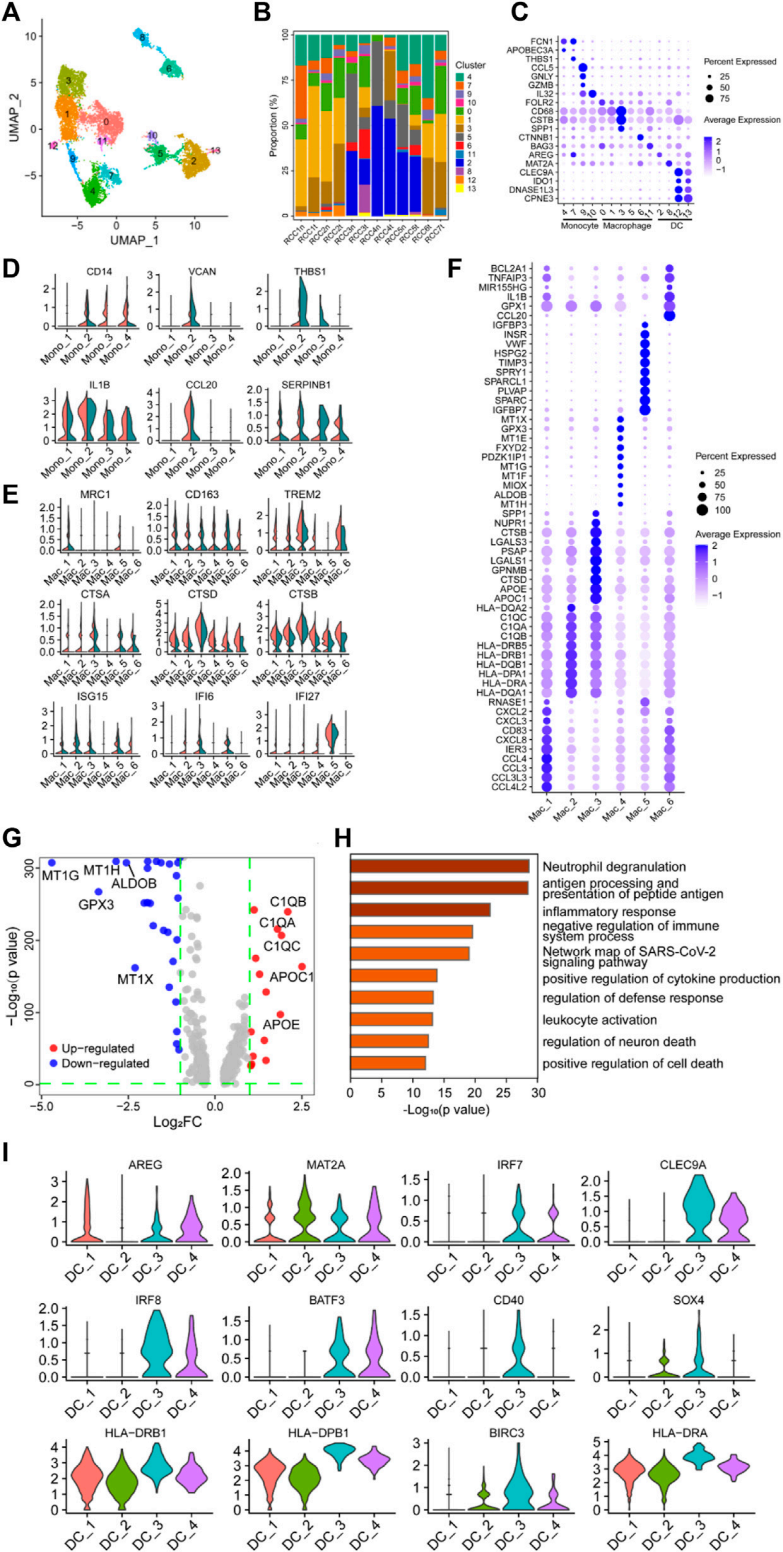
Myeloid cells have a negative immunoregulatory function in ccRCC. **(A)** UMAP plot of myeloid cells derived from ccRCC and para-tumor samples, colored according to clusters. **(B)** The proportion of 14 clusters of cells that originated from five para-tumor and seven tumor samples. **(C)** The cell-marker genes of three myeloid cell types identified in this study. **(D–E)** Violin plots of functional genes for **(D)** monocytes or **(E)** macrophages. Tumor tissues were colored red, while normal tissues were colored green. **(F)** Bubble chart showing the expression level of the top 10 differentially expressed genes (DEGs) in tumor-associated macrophages (TAMs). **(G)** Volcano plot of DEGs between TAMs and normal para-tumor macrophages. Upregulated genes [log_2_ (Fold Change) > 1] were indicated with red color, while downregulated genes [log_2_ (Fold Change) < −1] were indicated with blue. The top five upregulated and downregulated genes were annotated. **(H)** Gene ontology annotation of upregulated and downregulated DEGs. DEGs with FDR < 0.05 were considered significantly enriched. **(I)** Violin plots of functional genes for dendritic cells. Red represents tumor tissues while green represents normal tissues.

The myeloid cells in Figure 1B were re-clustered into 14 groups (Figure 3A; Supplementary Figure S4), and the proportion of these clusters in each sample is shown in Figure 3B. Subsequently, the 14 clusters of myeloid cells were classified into three major subgroups based on the expression of specific marker genes: monocytes (cluster 4/7/9/10), macrophages (cluster 0/1/3/5/6/11), and dendritic cells (DCs) (cluster 2/8/12/13) (Figure 3C).

#### 3.3.2 Tumor-infiltrating monocytes express genes that inhibit both immune and inflammatory responses in the TME

Differential expression analysis was performed on monocytes belonging to clusters 4/7/9/10, and the top 10 DEGs are shown in the heatmap, indicating that these four clusters may have latent heterogeneity in function (Supplementary Figure S4B). The expression levels of some immunoregulatory factors in monocytes were determined. VCAN, THBS1, and CCL20 were highly expressed in the Mono_2 cluster, which was derived from tumor tissue (Figure 3D), indicating the potential of this specific monocyte cluster to exert a critical immunosuppressive effect (Shearer et al., 2016; Xiao et al., 2018; Zhang et al., 2021b; Chen et al., 2021; Kellar et al., 2021; Wang et al., 2022a). Unexpectedly, CD14, IL1B, and SERPINB1 were detected in the Mono_2 cluster. The proteins encoded by these genes are involved in innate immune and inflammatory responses, indicating that these monocyte clusters have potential anti-tumor effects.

#### 3.3.3 Macrophages mainly exhibit M2 polarization in the TME of ccRCC

The single-cell transcriptome data revealed a highly heterogeneous expression pattern among macrophages in ccRCC, primarily indicating the prevalence of M2 phenotypes with a minor presence of M1 phenotypes. The top 10 DEGs in the selected clusters are shown in the bubble chart (Figure 3F). Several genes involved in cytokine pathways, including CCL3, CCL4, CXCL2, CXCL3, CXCL8, CCL3L3, and CCL4L2, were overexpressed in the Mac_1 cluster. Cluster Mac_2 showed elevated expression of genes related to MHC class II molecules (HLA-DRB1, HLA-DRB5, HLA-DQB1, HLA-DPA1, HLA-DRA, HLA-DQA1, and HLA-DQA2), suggesting a potential role in antigen presentation. The Mac_3 cluster displayed a high expression of GALS1, APOE, and APOC1, indicating involvement in anti-inflammatory functions (Abebayehu et al., 2017; Zheng et al., 2018; Chen et al., 2019; Lemos et al., 2019; Di Gregoli et al., 2020; Obradovic et al., 2021; Sherman, 2021; Suzuki et al., 2021; Chalise et al., 2022; Kang et al., 2022; Wen et al., 2022). The Mac_4 cluster demonstrated significant expression of MT1-related genes (MT1E, MT1F, MT1G, MT1H, and MT1X), which metallothioneins involved in immune regulation and the promotion of tolerogenic DCs with immunosuppressive functions (Subramanian Vignesh and Deepe, 2017; Wolf et al., 2020). Additionally, TIMP3, SPARC, IGFBP3, and IGFBP7 were highly expressed in the Mac_5 cluster, and these genes were involved in immunosuppression, tumor cell proliferation, and angiogenesis (Kielczewski et al., 2009; Pianta et al., 2015; Min et al., 2016; Talior-Volodarsky et al., 2017; Wang et al., 2021a; Rao et al., 2022). CCL20, GPX1, and BCL2A1 were highly expressed in the Mac_6 cluster. Compared with macrophages in the control group, MRC1, CD163, and TREM2 were highly expressed in TAMs, especially in Mac_4, 5, and 6 clusters, indicating a tendency of TAMs to polarize toward the M2 phenotype (Figure 3E). Furthermore, NR4A subfamily genes (NR4A1 and NR4A2) and Kruppel-like factor family genes (KLF2 and KLF4) were highly expressed in clusters Mac_4, 5, and 6 (Supplementary Figure S4C). Generally, heat shock protein family genes (HSPD1, HSPA1B, HSP90AB1, and HSPH1) enhance tumor growth and invasion through complex intracellular signaling networks. However, TAMs had similar or lower expression levels of these genes, especially HSPD1 and HSPH1, compared with the control clusters. Only the expression level of HSP90AB1 in the cluster Mac_6 was higher than that in the control cluster, suggesting potential pro-tumorigenic functions associated with EMT, tumor progression, metastasis (Wang et al., 2019; Jia et al., 2021), and immunotherapy resistance (Kosinsky et al., 2019). Additionally, interferon regulatory genes (ISG15, IFI6, and IFI27), the classical complement pathway (C1QA, C1QB, and C1QC), and cathepsin genes (CTSA, CTSB, and CTSD) were highly expressed in TAMs (Figures 3E, G). Studies have shown that these genes are associated with tumor-promoting properties, including tumor invasion, angiogenesis, inflammation inhibition, and metastasis (Vasiljeva et al., 2006; Bengsch et al., 2014; Akkari et al., 2016; Szekely et al., 2018; Roumenina et al., 2019; Park et al., 2020; Xu et al., 2021a; Wang et al., 2021b; Obradovic et al., 2021; Revel et al., 2022; Skopál et al., 2022). Based on functional gene expression, it was confirmed that these clusters had the function of the M2 phenotype. GO enrichment analysis showed that DEGs in the clusters were enriched in neutrophil degranulation, antigen presentation, inflammatory response, and negative regulation of the immune system process (Figure 3C. These findings collectively indicated that TAMs played a role in negatively regulating immune responses in the context of ccRCC.

#### 3.3.4 The function of dendritic cells was complex and heterogeneous

DCs are the major antigen-presenting cells in the TME. Re-clustering analysis followed by differential expression analysis of the clustered cells identified a population of heterogeneous DCs, with either antitumor or pro-tumor potential, among myeloid cells (Figure 3C). The expression of some genes was visualized using violin plots (Figure 3I, Supplementary Figure S4D), and their expression profiles showed marked heterogeneity across the four clusters of DC subpopulations. Genes involved in MHC II-restricted antigen presentation (HLA-DRB1, HLA-DPB1, HLA-DRA, HLA-DQA1, HLA-DQB1, HLA-DRB5, and HLA-DPA1) were highly expressed in clusters DC_3 and DC_4 compared to other DCs clusters in the TME. BATF3, CLEC9A, and IRF8 were highly expressed among MHC-II gene-expressing cells, especially in cluster DC_3, implying that this cluster was a type I conventional DCs (CD40, BATF3, CLEC9A, IRF8, and AREG), which may be involved in activating antitumor T cell inflammatory immune responses (Seillet et al., 2013; Pires et al., 2019; Rojahn et al., 2020; Tullett et al., 2020; Xu et al., 2021b; Zhang et al., 2021c; Gou et al., 2021; Hongo et al., 2021; Kim et al., 2021; Anderson et al., 2022).

The correlation between the abundance of DCs and T cells in tumor samples from the TCGA-KIRC database was determined using the MCPcounter algorithm. The abundance of DCs was significantly positively correlated with the abundance of T cells and cytotoxic lymphocytes in tumor tissues (Supplementary Figures S5A, B), partly validating the findings of the scRNA sequencing data analysis. The subpopulation of cells with high expression of BIRC3 was involved in tumor suppressive regulatory role (Andersen et al., 2017; Nakamizo et al., 2021), while IRF7 recruited activated inflammatory cells producing interferons (Tomasello et al., 2018; Zhang et al., 2021d; Somebang et al., 2021). Paradoxically, MAT2A and SOX4 were highly expressed in this cluster. MAT2A acted as a tumor-protective factor, protecting tumor cells from ferroptosis and promoting growth (Liu et al., 2021a; Villa et al., 2021; Ma et al., 2022). SOX4 was associated with myeloid cell development, apoptosis, and tumorigenesis (Das et al., 2017; Renosi et al., 2021), indicating that tumor-associated DCs were functionally heterogeneous.

The top 10 DEGs between the tumor-associated DCs and control DCs are illustrated using bubble plots (Supplementary Figure S4E). Highly expressed ribosomal protein-encoding genes in cluster DC_4 exhibited tumor-promoting or tumor-suppressing effects. Tumor-suppressing gene sets (RPL4, RPLP1, RPL18A, RPL32, RPL13, RPL39, RPL37, RPS2, and RPS27) suppressed tumor protein synthesis, enforced p53 signaling, induced tumor cell senescence, reduced tumor cell viability and proliferation, and inhibited tumor development (Xiong et al., 2011; Kardos et al., 2014; Cho et al., 2020). In contrast, tumor-promoting gene sets (RPS23, RPS11, RPS8, RPS3A, RPSA, and RPS15A) could promote tumor progression by suppressing the expression levels of inflammatory and tumor necrosis factors, alleviating immune infiltration and TME inflammatory responses, and activating oncogenic signaling pathways (Zhou et al., 2020a; Zhou et al., 2020b; Sun et al., 2020; Liu et al., 2021b; Liu et al., 2022).

Overall, these results indicated that cluster DC_4 exerted opposite effects in different TMEs. Similarly, highly expressed genes (CD40, BATF3, CLEC9A, IRF8, and AREG) among cluster DC_3 cells exhibiting type I conventional DC phenotype were functionally and diametrically opposite (Supplementary Figure S4E). Although the cells exhibited an antitumor phenotype, they expressed pro-tumorigenic factors (TXN and S100A10). TXN mediates the elimination of reactive oxygen species, protects the cell membrane structure, and induces radiotherapy resistance in malignant cells (Yu et al., 2022). S100A10 is involved in macrophage infiltration into tumor tissues, development of drug resistance by tumor cells during clinical chemotherapy (Li et al., 2021a), and invasion and metastasis of malignant tumors (Li et al., 2021a). In contrast, SNX3 and LGALS2 are antitumor factors that inhibit tumor cell growth and metastasis (Li et al., 2021b; Yu et al., 2022), which is consistent with the phenotype of type I conventional DCs. Cluster DC_1 and cluster DC_2, representing the other two cell subgroups, showed no distinct tumor immune functions, possibly indicating a quiescent or suppressive state within the TME.

### 3.4 CD8^+^ T-cells were involved in heterogeneous immune functions in the ccRCC TME

**FIGURE 4:**
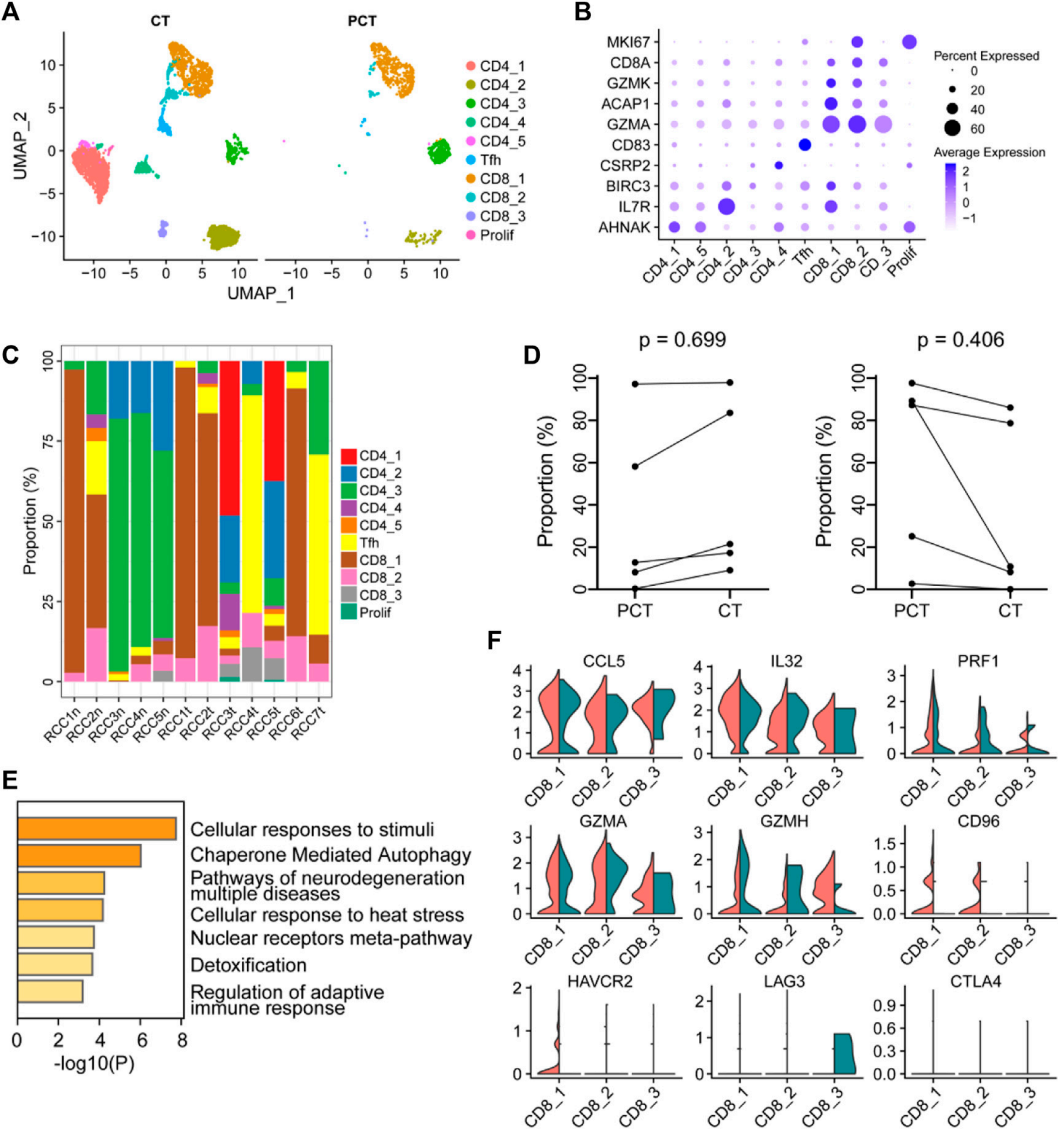
The immune function of CD8^+^ T-cells in the ccRCC tumor microenvironment is heterogeneous. **(A)** UMAP plot of T lymphocytes derived from ccRCC and the para-tumor samples, colored according to cluster and cell type. **(B)** The cell-marker genes of the lymphocyte types identified in this study. **(C)** The fraction of 10 clusters of T lymphocytes that originated from the five para-tumor and seven tumor samples. **(D)** The proportion of CD4^+^ (left) or CD8^+^ (right) cells to total T lymphocytes was measured. Paired *t*-test was performed using GraphPad Prism 5. *p* < 0.05 was considered statistically significant. **(E)** Gene ontology analysis of differentially expressed genes (DEGs) in CD8^+^ T cells between ccRCC and para-tumor samples. DEGs with FDR < 0.05 were considered significantly enriched. **(F)** Violin plots of functional genes for CD8^+^ T cells. Red represents tumor tissues while green represents normal tissues.

T cells in the tumor and para-tumor tissues were re-clustered into four subgroups based on specific marker genes (Figures 4A, B). The proportion of each subgroup of cells in the different samples is shown in Figure 4C. CD4_1 and CD4_5 cell clusters were significantly enriched in tumor tissues, suggesting their potential involvement in antitumor cytotoxicity (Oh et al., 2020; Sacher et al., 2020). However, there was no significant difference in the expression of CD4^+^ T cells and CD8^+^ T cells between the tumor and para-tumor tissues. The proportion of CD4^+^ T cells displayed an increasing trend, while the proportion of CD8^+^ T cells showed a decreasing trend in tumor samples (Figure 4D). GO functional enrichment analysis revealed that highly expressed genes in CD8^+^ T cells derived from ccRCC were enriched in cell response to stimulation and regulation of the adaptive immune response (Figure 4E). Using bulk RNA-sequencing data from the public database, we also found that CD8^+^ T cells were associated with immune regulation in renal cancer samples. GSVA of the sequencing data from TCGA-KIRC database showed that the abundance of CD8^+^T cells was positively correlated with regulation of adaptive immune response and regulation of T-cell costimulation (Supplementary Figures S5C, D). Additionally, the expression levels of some immune factors in the cells are shown in Figure 4F. Furthermore, CTLA4 and LAG3 were negatively expressed in tumor-infiltrating lymphocytes, while CD96 and HAVCR2 were highly expressed in cluster CD8_1, indicating functional exhaustion of cells in this cluster within TME. Additionally, the low expression of PRF1 (a class of cytotoxic T molecules) and GZMH in cluster CD8_1 cells also confirmed this phenotype. Compared with para-tumor-derived CD8^+^ T cells, immune checkpoint genes (LAG3, CTLA4, CD96, and HAVCR2) were under-expressed in tumor-derived CD8^+^ T cells, while cytotoxic effector molecules (GZMH and GZMA) and pro-inflammatory cytokines (IL32 and CCL5) were highly expressed, indicating that these cells in cluster CD8_3 were tumor-associated cytotoxic T lymphocytes. Low expression of immune checkpoint molecules in cluster CD8_3 of CD8^+^ T cells in the TME may result in low risk of early disease progression and non-aggressive histological properties in ccRCC (Ballesteros et al., 2021).

### 3.5 Two functionally distinct cancer-associated fibroblasts lineages in ccRCC

**FIGURE 5:**
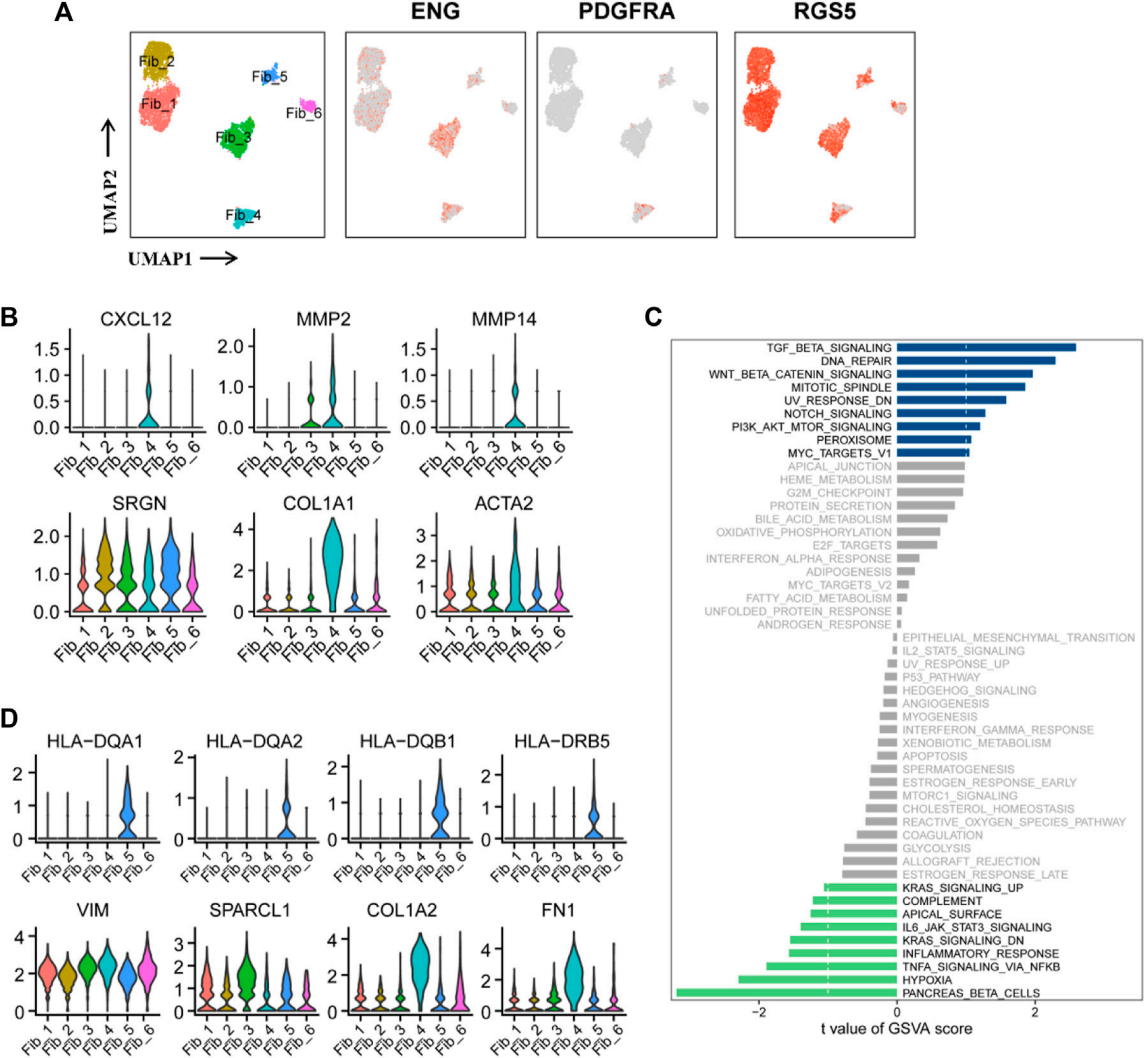
Two functionally distinct cancer-associated fibroblasts lineages in ccRCC. **(A)** UMAP plot of the cancer-associated fibroblasts derived from the ccRCC samples, colored according to cluster and subgroup markers (ENG, PDGFRA, and RGS5). **(B)** Violin plots of marker genes for six clusters of CAFs. **(C)** GSVA scores between ENG^high^ and ENG^low^ CAFs were quantified based on *t* value and visualized using histogram. *t* < −1 was considered significantly enriched in the ENG^low^ CAFs, and *t* > 1 as significantly enriched in the ENG^high^ CAFs. **(D)** Violin plots of marker genes and MHC-II molecular genes in six clusters of CAFs.

Cancer-associated fibroblasts (CAFs) in Figure 1B were re-clustered into six subpopulations. The expression of the three marker genes in CAFs is shown in Figure 5A. PDGFRA serves as a marker for inflammatory-associated fibroblasts (iCAFs), while RGS5 is associated with the development of myofibroblasts (mCAFs). Cluster Fib_4, with a small cell population, likely represents iCAFs, while the majority of CAFs may possess mCAFs potential (Figure 5A). ENG expression in fibroblasts has been linked to two distinct fibroblast lineages with contrasting functions. ENG^high^ fibroblasts promote tumor cell growth, whereas ENG^low^ fibroblasts have a strong tumor inhibition effect (Hutton et al., 2021). Therefore, we investigated the role of CAFs in the tumor stroma based on ENG expression. Differential expression analysis was performed between ENG^high^ CAFs (clusters Fib_3 and 4) and ENG^low^ CAFs (cluster Fib_1/2/5/6), and the top 10 DEGs are shown in a bubble chart (Supplementary Figure S6A). Cluster Fib_5 prominently expressed MHC-II genes (HLA-DQA1, HLA-DQA2, HLA-DQB1, HLA-DRB5, HLA-DRB1, HLA-DRA, HLA-DPA1, and HLA-DPB1), indicating their role as antigen-presenting cells with tumor-suppressing effects. Gene signatures (VIM, SPARCL1, COL1A1, and FN1) and canonical fibroblast markers confirmed their fibroblast identity (Figure 5D; Supplementary Figure S6A). Cluster Fib_6 cells exhibited high expression of MT1-related genes (MT1E and MT1X) that activate tumor-suppressing immune cells (Supplementary Figure S6A). GSVA analysis showed substantial variation in multiple Hallmark pathways among fibroblast subgroup (Supplementary Figure S6B).

Notably, these findings have been partially verified in ccRCC samples. GSVA of the sequencing data from TCGA-KIRC database showed that the abundance of fibroblasts was positively correlated with INFLAMMATORY_RESPONSE, RESPONSE_TO_ROS, APICAL_JUNCTION_ASSEMBLY, REGULATION_OF_SPROUTING_ANGI OGENESIS, REGULATION_OF_CELL_CYCLE_CHECKPOINT, and REGULATION_OF_DNA_REPAIR (Supplementary Figure S7). Subsequently, comparison of GSVA scores between ENG^high^ and ENG^low^ CAFs revealed enrichment of cancer-promoting signaling pathways in ENG^high^ cells, including TGFβ, DNA repair, PI3K/AKT/mTOR, and Wnt/β-catenin, while cancer-suppressive pathways, such as hypoxia, IL6/JAK/STAT3, TNF-α, and inflammatory response, were enriched in ENG^low^ cells (Figure 5C). These findings confirm the significant role of ENG in classifying CAFs as tumor-promoting or tumor-suppressing. Notably, cluster Fib_4 cells exhibited tumor-promoting potential as they overexpressed CXCL12, MMP2, and MMP12. CXCL12 is an inhibitory factor associated with reduced macrophage activation, inhibition of CD25 expression, and T-cell proliferation (Walterskirchen et al., 2022). MMP2 and MMP12 increase tumor malignancy and promote tumor metastasis and progression. SRGN, COL1A1, and ACTA2 were the three fibroblast markers (Figure 5B). Overall, ENG^high^ CAFs exhibited tumor-beneficial effects (Figure 5C).

### 3.6 Single-cell atlas of endothelial cell phenotypes in ccRCC and adjacent tissues

**FIGURE 6:**
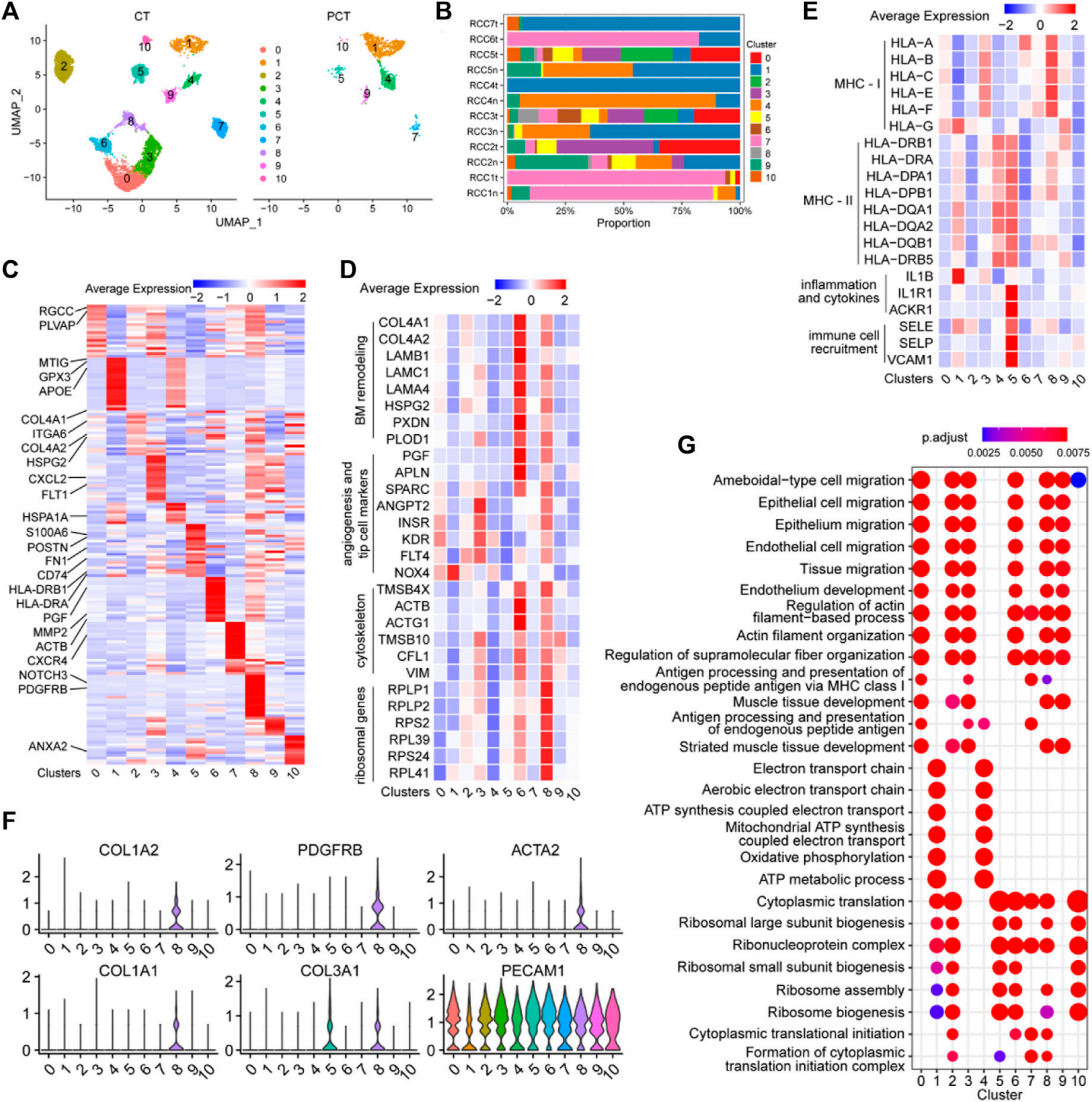
Single-cell atlas of endothelial cell phenotypes in ccRCC and adjacent tissues. **(A)** UMAP plot of the endothelial cells derived from cancer tissues and para-cancer tissues, colored according to cluster. **(B)** The fraction of 11 clusters of endothelial cells that originated from five para-tumor and seven tumor samples. **(C)** Heatmap showing differentially expressed EC-enriched genes between ECs in tumor and para-tumor tissues. **(D)** The expression levels of selected marker genes (ribosomal genes, cytoskeleton, angiogenesis and tip cell markers, BM remodeling) of 11 clusters of ECs are illustrated in a heatmap. **(E)** The expression levels of selected marker genes (MHC-I, MHC-II, inflammation and cytokines, immune cell recruitment) of 11 clusters of ECs are displayed in a heatmap. **(F)** Violin plots of functional genes of ECs the ccRCC tissues. **(G)** Significantly enriched GO term by DEGs in 11 clusters of ECs in ccRCC tissues.

The ECs in Figure 1B were further analyzed and re-clustered into 11 subgroups (cluster 0–10) (Figure 6A). The proportion of each EC cluster in each tissue sample varied considerably (Figure 6B). Differential expression analysis of ECs between tumor and adjacent normal tissues, revealed the top 20 DEGs for each cluster, visualized in a heatmap (Figure 6C). The heatmap specifically indicated important marker genes of the EC clusters.

Cluster 0 ECs may be involved in immune response and angiogenesis in the TME. Single-cell transcriptome data of this cluster reflected the high expression levels of RGCC, a gene associated with complement activation and apoptosis (Voigt et al., 2019), and PLVAP, a gene associated with angiogenesis (Wang et al., 2021c). The role of ECs in cluster 0 was verified (Figure 6D), as evidenced by the high expression of angiogenic sprouting genes (KDR, INSR, ANGPT2, NOX4, PTP4A3, and FLT4) in cluster 0 ECs (Figure 6E). The inflammatory regulatory genes MT1G, GPX3, and IL1B were highly expressed in cluster 1 ECs (Figures 6C, E), indicating that these cells may participate in the regulation of immune responses in the TME. Cluster 2 ECs may be involved in extracellular matrix (ECM) remodeling in ccRCC, as evidenced by the high expression of ECM protein-coding genes (COL4A1, COL4A2, HSPG2, and ITGA6) in this subgroup (Figure 6C). Angiogenesis-related genes (FLT1, KDR, SPARC, INSR, ANGPT2, and FLT4) and MHC-I molecules (HLA-A, HLA-B, HLA-C, HLA-E, and HLA-F) were highly expressed in cluster 3 ECs, indicating that the cells may function as antigen-presenting cells and promote tumor parenchymal angiogenesis (Figures 6C, E). Additionally, the cytokine marker gene CXCL2 was also significantly upregulated in cluster 3 ECs, indicating that the cells may also be involved in tumor-related inflammation. MHC-II molecules (HLA-DRB1, HLA-DRA, HLA-DPA1, HLA-DPB1, HAL-DQA1, HAL-DQA2, HAL-DQB1, and HLA-DRB5) were highly expressed in cluster 4 ECs, indicating that the cells may be involved in antigen presentation (Figure 6E). Additionally, HSPA1A, a marker of endothelial cell activation (Liu et al., 2008), was overexpressed in cluster 4 ECs, indicating that cells are a class of activated ECs (Figure 6C). Sequencing data showed that endothelial-mesenchymal transition genes (FN1 and POSTN), MHC-II antigen-presenting molecule genes (HLA-DRB1, HLA-DRA, HLA-DPA1, HLA-DPB1, HAL-DQA1, HAL-DQA2, HAL-DQB1, HAL-DRB5, and CD74), and immune response activation-related genes (IL1E1, ACKR1, SELE, SELP, and VACM1) were significantly upregulated in cluster 5 ECs, indicating that the cells have varied and complex functions. Moreover, these gene sets were closely associated with inflammatory responses and immune cell recruitment (Figures 6C, E). The upregulation of these signature genes indicated that cluster 5 ECs are the primary effector cells of anti-tumor immunity in the TME.

Cluster 6 ECs may be involved in mediating tumor vascular growth and basement membrane (BM) remodeling, as evidenced by the upregulation of BM remodeling genes (COL4A1, COL4A2, LAMB1, LAMC1, LAMA4, HSPG2, PXDN, PLOD1, and MMP2), angiogenesis-related genes (PGF, APLN, and SPARC), and cytoskeletal genes (TMSB4X, ACTB, ACTG1, TMSB10, CFLS, and VIM) in cluster 6 ECs (Figures 6C, D). CXCR4 was highly expressed in cluster 7 ECs (Figure 6C), and this chemokine receptor binds to CXCL12-expressing TAMs and promotes tumor metastasis and progression (Mota et al., 2016), indicating the pro-cancer properties of this cell cluster. Vascular wall marker molecules (PDGFRB and NOTCH3) were significantly expressed in cluster 8 ECs, suggesting that this cluster may belong to a group of vascular wall ECs (Figure 6C). Additionally, ribosomal protein-encoding genes (RPLP1, RPLP2, RPS2, RPL39, RPS24, and RPL41), cytoskeleton-related genes (TMSB4X, ACTB, ACTG1, TMSB10, CFL, and VIM), and some angiogenesis-related genes were upregulated in cluster 8 ECs (Figure 6D), indicating that the cells may be involved in promoting tumor growth and protein synthesis. Moreover, PECAM1^+^ cells (ECs) in cluster 8 ECS (COL1A1, COL1A2, COL3A1, PDGFRB, and ACTA2) showed an endothelial-mesenchymal transformation phenotype (Figure 6F), which contributed to tumor progression. Interestingly, this population of cells may also be involved in tumor antigen presentation, since genes for MHC-I molecules (HLA-A, HLA-B, HLA-C, HLA-E, and HLA-F) were highly expressed in this cluster (Figure 6E). TMSB10 was highly upregulated in cluster 9 ECs, and is involved in facilitating the expression of VEGF signaling factors (Zhang et al., 2018), indicating that cluster 9 ECs may be related to the promotion of cytoskeleton formation and tumor angiogenesis in tumor tissues. ANXA2 was upregulated in cluster 10 ECs, and is involved in increasing the permeability between ECs by reducing the expression of inter-endothelial binding proteins (Li et al., 2019), which is conducive to tumor metastasis. These findings provide detailed insights into the functional characteristics of EC subgroups within TME and enhance our understanding of the complex interplay between ECs and tumor biology, highlighting potential targets for therapeutic intervention.

GO enrichment analysis of DEGs in each EC cluster between tumor and control tissues was performed to elucidate the function of each group of ECs. Cell migration pathways were highly enriched in clusters 0/2/3/6/8/9 cells, and antigen presentation function was mainly enriched in clusters 0/3/7 (Figure 6G). Energy metabolism-related pathways (oxidative phosphorylation, ATP metabolic process, and electron transport chain) were mainly enriched in clusters 1 and 4. Protein synthesis pathways (ribosomal subunit biosynthesis, cytoplasmic translation, and ribosomal assembly) were enriched in clusters 1/2/5, 6/7/8, and 10. These findings shed light on the diverse functional characteristics of EC clusters in the tumor microenvironment.

## 4 Discussion

The immune microenvironment of ccRCC is highly complex and characterized by significant immune infiltration, making it challenging to fully characterize the heterogeneity within the tumor (Hu et al., 2020; Krishna et al., 2021). The application of multi-omics techniques to explore the heterogeneity of components within the ccRCC microenvironment is a common and effective approach in current tumor research. Utilizing multidimensional information at the single-cell level to address ccRCC heterogeneity offers new insights into tumor regulatory mechanisms and identification of potential therapeutic targets. Methods for studying tumor heterogeneity include single-nucleus RNA sequencing (snRNA-seq), single-cell assay for transposase-accessible chromatin using sequencing (scATAC-seq), single-cell sequencing, and T-cell receptor (TCR) sequencing, among others, enabling the analysis of intratumoral heterogeneity at the single-cell level.

Tumor cells constitute only a small fraction (7.2%) of all cells in ccRCC tissue, and traditional bulk epigenetic sequencing methods may fail to identify tumor cell-specific regulatory elements and their networks (Young et al., 2018). ScATAC-seq, facilitated by Tn5 transposase-mediated labeling, identifies accessible chromatin regions and captures active DNA regulatory elements at single-cell resolution (Buenrostro et al., 2015). Long et al. applied scRNA-seq and scATAC-seq to generate transcriptional and epigenomic landscapes of ccRCC, revealing genetic instability and increased methylation as adverse prognostic markers that exhibit heterogeneity across renal cancer samples (Long et al., 2022). This method captures diverse types of gene regulatory information. The combination of these two methods allows the identification of genome-wide cis-regulatory elements and inference of transcription factor (TF) binding and activity at the single-cell level (Chiou et al., 2021). By conducting multi-omics analysis of primary tumor tissues from ccRCC, key transcriptional molecules that mediate tumor development and manipulate immune cell function can be identified, facilitating the exploration of upstream regulatory targets (Young et al., 2018; Muto et al., 2021; Long et al., 2022). In addition, the combination of scRNA-seq and TCR sequencing enables the exploration of transcriptional heterogeneity in tumor tissues and immune cells in the blood of cancer patients. Studies have shown that CD8^+^ T cells and macrophages in tumor-infiltrating immune cells are overall increased compared to normal renal tissue, and these tumor-infiltrating immune cells exhibit distinct cellular transcriptional states and activation statuses. This provides an advantage over traditional methods that rely on targeting known immune cell components, such as flow cytometry and immunohistochemistry, allowing the identification and characterization of new immune cell subpopulations. The transcriptional landscape of T lymphocytes, combined with TCR sequencing, provides unprecedented depth in measuring the clonal T-cell response to cancer (Borcherding et al., 2021). The integration of scATAC-seq, scRNA-seq, and whole-exome sequencing can be used to understand heterogeneity between individuals and construct single-cell transcriptomic and chromatin accessibility maps of ccRCC, thereby revealing the regulatory features of different tumor cell subtypes (Yu et al., 2023).

However, most methods for assessing intratumoral heterogeneity are also limited by two-dimensional *ex vivo* tissue analysis. Dynamic contrast-enhanced magnetic resonance imaging (DCE-MRI) can assess the spatial landscape of the entire tumor within its *in vivo* environment. When combined with vertically integrated radiogenomic co-localization methods, it can be used for multi-region tissue collection and analysis, determining the radiographic differences in tumor sections that exhibit transcriptional heterogeneity. This approach helps integrate RNA sequencing data from multi-region tumor samples with DCE-MRI enhancement information in tumor spatially co-localized regions and can be utilized to assess the clinical applicability of anti-tumor targeted therapy in metastatic RCC patients (Udayakumar et al., 2021). Deep multi-region whole-exome and transcriptome sequencing of patients before and after treatment, combined with monitoring of T-cell repertoires in tissue and peripheral blood, can analyze the temporal and spatial variations in genomic and immune phenotypic features of ccRCC patients, revealing the underlying reasons for differential responses following immune checkpoint inhibitor (ICI) treatment. Studies have shown that increased intratumoral heterogeneity (ITH) is associated with a range of genomic features, such as CDKN2A/B loss, and microenvironmental features, including elevated myeloid lineage expression, decreased peripheral TCR diversity, and neoantigen depletion. ITH further impacts patients’ response to ICI and targeted therapies. This contributes to the development of clinically meaningful biomarkers and highlights important features of tumor evolution under ICI treatment (Golkaram et al., 2022).

Currently, there is no effective treatment for RCC, especially for metastatic RCC. Although PD-1 blockade combined with AKI inhibitors is currently the focus of immunotherapy for renal cancer, the overall response rate ranges from 37%–58% (Grimm et al., 2019; Kotecha et al., 2019; Hu et al., 2020). Therefore, studies are necessary to identify therapeutic targets for renal cancer treatment to prolong the survival time of patients. In the present study, we performed bioinformatic analysis of single-cell sequencing dataset (GSE156632) to elucidate the intratumoral heterogeneity in the ccRCC TME. A total of 11 different cell types were identified; however, we focused on five cell types: tumor cells (proximal tubule cells), myeloid cells, T lymphocytes, fibroblasts, and ECs. Additionally, an independent single-cell sequencing dataset and bulk RNA-sequencing data from TCGA database were used to partially verify the results.

CNV analysis and CNV scoring were performed on renal carcinoma cells and normal proximal tubule cells, revealing a higher occurrence of CNVs in tumor cells compared to normal tubule cells (Figures 2D, E). The CNVs were considered to have potential biological significance. For instance, the gain or deletion of copy numbers in chromosomes can affect the biological behavior and metabolism of tumor cells. Loss of chromosome 3p results in upregulation of hypoxia signaling, downregulation of glycolysis and oxidative phosphorylation (OXPHOS), and changes in the cell cycle, and is associated with fatty acid metabolism and the TCA cycle (Adashek et al., 2020; Jonasch et al., 2021). Gain of chromosome 5q results in the upregulation of mTORC1 and MYC signals (Li et al., 2013; Benstead-Hume et al., 2019). Additionally, deletion of chromosome 3p and gain of 5q are thought to be early changes in ccRCC development (Yoshikawa et al., 2022). Moreover, the gain of 6p is associated with higher tumor grades, advanced tumor stages, and upregulation of the TFEB protein (Williamson et al., 2017). Gain of 7p has been reported to promote protein translation and EMT. VGEFR and IGBP3 are also located on chromosome 7p, which contributes to the aggressive phenotype of cancer cells (Pezzolo et al., 2009; Cimino et al., 2018; Fernandes et al., 2021). Several studies have shown that loss of 9p can lead to the deletion of the tumor suppressor CDKN2A, which is related to the upregulation of translation initiation, mTOR, and MYC signals (Baietti et al., 2021; Yi et al., 2022; Yoshikawa et al., 2022). Moreover, loss of 14q induced the upregulation of MYC signaling, N-glycosylation, and the IFN-γ signaling pathway in tumor cells, and was identified in 75% of CIMP^+^ tumors. CIMP^+^ tumors have increased malignancy, including enhanced MYC signaling and protein translation, and unique characteristics associated with increased OXPHOS and reduced adhesion plaques (Park et al., 2019). Furthermore, CNV analysis on the scRNA-seq validation dataset further supported the heterogeneous nature of CNVs observed in the scRNA-seq data. The CNVs in tumor cells are closely related to the pathogenesis of ccRCC and may be a potential source of new diagnostic, prognostic, and therapeutic biomarkers (Fernandes et al., 2021).

Based on the CNV score of renal carcinoma cells, the tumor cells were classified into high and low CNV groups. Differential expression analysis showed that MALAT1 was upregulated in both groups (Figure 2G). MALAT1, a long non-coding RNA, was found to activate AMPK signaling, promoting cancer cell proliferation (Wang et al., 2021d). Moreover, MALAT1 upregulated the expression of CD36 and liposynthases (PPARγ, PGC1α, SREBP-1C, FAS, and ACC), enhancing unsaturated fatty acid synthesis and uptake, thereby promoting tumor cell progression (Huangfu et al., 2018; Ebrahimi et al., 2020; Wang et al., 2021d). TCGA data showed that high MALAT1 expression was associated with poor prognosis in RCC patients (Figure 2I). Subsequently, GSVA revealed high heterogeneity in the expression of signaling pathways in both the high and low CNV groups of renal carcinoma cells. Epithelial-mesenchymal transition, angiogenesis pathway, Notch signaling pathway, and inflammatory response were upregulated in the low CNV group, but downregulated in the high CNV group. Conversely, mTORC signaling, interferon-alpha response, the reactive oxygen species pathway, E2F targets, DNA repair, and the p53 pathway were upregulated in the high CNV group, but downregulated in the low CNV group (Figure 2H). Overall, these results confirmed that renal cancer cells are highly heterogeneous in terms of gene expression and biological behavior, which could contribute drug resistance in neoadjuvant therapy (Hu et al., 2020).

Additionally, analysis of myeloid cells indicated three cell types: Monocytes, macrophages, and DCs, and each cell type consisted of several clusters with different functions. Among the monocyte clusters, Mono_2 cluster derived from the tumor tissue (VCAN, THBS1, and CCL20) was found to play an important immunosuppressive role (Figure 3D). For instance, VCAN can inhibit the recruitment of monocytes and neutrophils to tumor tissues, thereby reducing the anti-tumor inflammatory response (Kellar et al., 2021). The proteins encoded by CD14, IL1B, and SERPINB1 participate in the innate immune and inflammatory responses, indicating that this monocyte cluster have potential antitumor effects (Gong et al., 2011; Burgener et al., 2019; Kim et al., 2022; Kowalska et al., 2022). On the whole, monocytes exhibit a prevailing immunosuppressive phenotype, aligning with previous investigations. In concurrence, Kim et al. identified a propensity of monocytes to undergo a phenotypic shift towards an anti-inflammatory state in the context of metastatic lung adenocarcinoma, thereby facilitating the establishment of an inhibitory immune microenvironment (Kim et al., 2020). Similarly, Xu et al. observed impaired monocyte differentiation within the tumor microenvironment of gastric cancer, whereby these cells engage in intercellular communication with tumor stromal cells or neoplastic cells, promoting tumor progression (Xu W. et al., 2022). Therefore, the activation of monocytes via immunotherapy could be a promising treatment strategy.

Macrophage clusters were comprehensively analyzed, revealing their distinct phenotypic profiles. Macrophages exhibit polarization towards either M1 or M2 phenotypes in response to inflammatory cues. Apart from the Mac_1 cluster, the remaining macrophage clusters predominantly display M2-like functions. Studies have associated intratumoral infiltration of M2-polarized TAMs with unfavorable clinical outcomes and depleted T-lymphocyte infiltration (Dannenmann et al., 2013; Giraldo et al., 2015). Our investigation identified a subset of CD163^+^ and TREM2^+^ TAMs, indicative of their inclination towards the M2 phenotype. Remarkably, these TAMs exhibited high expression of interferon regulatory genes (ISG15, IFI6, and IFI27) and cathepsin genes (CTSA, CTSB, and CTSD), known to contribute to pro-tumor properties such as invasion, angiogenesis, immune suppression, and metastasis (Vasiljeva et al., 2006; Bengsch et al., 2014; Akkari et al., 2016; Szekely et al., 2018; Roumenina et al., 2019; Park et al., 2020; Xu et al., 2021b; Wang et al., 2021b; Obradovic et al., 2021; Revel et al., 2022; Skopál et al., 2022). The Mac_6 cluster displayed elevated expression of NR4A subfamily genes (NR4A1 and NR4A2), Kruppel-like factor family genes (KLF2 and KLF4), and heat shock protein family genes (HSPD1, HSPA1B, and HSP90AB1), signifying their crucial role as M2-like macrophages. NR4A1 influences tumor cell proliferation (Guo et al., 2021), while NR4A2 promotes M2 polarization (Mahajan et al., 2015; Miao et al., 2022). KLF2 and KLF4 possess anti-inflammatory effects but also impact macrophage proliferation, differentiation, activation, and tumor growth inhibition, demonstrating their dual nature in tumor immunity (Zappasodi et al., 2015; Tian et al., 2021). Additionally, the heat shock protein family genes (HSPD1, HSPA1B, and HSP90AB1) facilitate tumor growth and invasion via intricate intracellular signaling networks (Roberts et al., 2017; Wang et al., 2021e). Previous research has linked these genes to tumor-promoting traits, including tumor invasion, angiogenesis, inflammation suppression, and metastasis. The functional gene expression patterns confirm the M2 phenotype features of these clusters. Consistent with previous single-cell studies, tumors are typically enriched with both M1 and M2-like macrophages, with a predominance of M2-polarized macrophages and an imbalance between pro-inflammatory M1-like and anti-inflammatory M2-like macrophages associated with disease progression (Hu et al., 2020; Braun et al., 2021; Dinh et al., 2021; Wang et al., 2022b). Similar to Wang et al. 2022b findings in thyroid cancer, our study reveals that besides expressing typical M2 markers, TAMs also exhibit elevated levels of several cathepsin proteases (CTSD, CTSL, and CTSB), further underscoring the potential association of these M2-like macrophages with tumor invasion, migration, and their relevance to targeted therapies. Collectively, these findings confirm the promotion of EMT, attenuation of interstitial inflammatory responses, tumor progression, metastasis (Wang et al., 2019; Jia et al., 2021), and induction of immunotherapy resistance (Kosinsky et al., 2019) by M2 macrophages through activation of proto-oncogenic signaling pathways in ccRCC. Furthermore, gene ontology enrichment analysis underscores the vital role of tumor-infiltrating macrophages in regulating immune responses and sustaining tumor cell survival. This study contributes to a comprehensive understanding of macrophage heterogeneity and M2 polarization in ccRCC, shedding light on potential therapeutic targets.

DCs play a pivotal role as major antigen-presenting cells within the TME. Our investigations have shed light on the heterogeneous nature of DCs in TME, highlighting the existence of distinct subpopulations with both anti-tumor and pro-tumor potentials. Gene expression analyses have revealed significant heterogeneity among different DC subgroups, particularly a group of type I conventional DCs (CD40, BATF3, CLEC9A, IRF8, and AREG) had a high expression of MHC-II molecules and tumor suppressors, indicating that these cells may be involved in activating adaptive immune response and inhibiting neoplasm. Furthermore, the abundance of DCs in tumor samples positively correlates with the presence of T cells and cytotoxic lymphocytes. While specific DC subgroups exhibit tumor-inhibitory regulatory functions, others may be involved in tumor promotion. Notably, a subset of DCs in a particular subgroup exhibits high expression of ribosomal protein genes, partitioned into gene sets associated with either pro-tumor or anti-tumor activities. Collectively, these findings underscore the heterogeneity of DCs in the TME and their distinct roles in tumor immunity. In the context of ccRCC, DCs demonstrate evident diversification. Similar observations have been made by Dinh et al. (2021) in the context of esophageal squamous cell carcinoma, wherein DCs exhibit multiple subtypes, with a majority presenting classical DC markers and a minority exhibiting immature markers. The presence of heterogeneous DCs in the TME has also been identified in hepatocellular carcinoma, where a considerable proportion of DCs expressing antigen-presenting genes has been observed (Sun et al., 2021). These results collectively indicate the heterogeneity of DCs in the TME and their potential for exerting anti-tumor effects.

Further analysis of T lymphocytes indicated the presence of CD96^+^HAVCR2^+^CD8^+^ T cells, namely, exhausted T lymphocytes, among CD8^+^ T cells. These immune cells experience immune dysfunction due to the activation of immune checkpoint pathways, which is positively correlated with adverse prognosis in cancer patients (Drake and Stein, 2018; Ballesteros et al., 2021). To overcome T-cell dysfunction and restore antitumor activity, adjuvant therapies targeting the TME and immune checkpoints are being investigated (Giraldo et al., 2017). Interestingly, not all CD8^+^ T-infiltrating lymphocytes were exhausted and dysfunctional. Cytotoxic effector molecules (GZMB and GZMA) and pro-inflammatory cytokines (IL32 and CCL5) were highly expressed in a special class of CD8^+^ T cells (LAG3^-^, CTLA4^-^, CD96^−^, and HAVCR2^-^), indicating that the cells were immune-activated tumor-infiltrating lymphocytes (TILs) and their abundance is beneficial for suppressing aggressive neoplasias. Various clusters of CD8^+^ T cells in the TME showed distinctive functional phenotypes, suggesting that the density and phenotype of TILs could predict both patient prognosis and clinical response to diverse adjuvant therapies (Giraldo et al., 2015; Becht et al., 2016; Ballesteros et al., 2021).

Among cancer-associated fibroblasts, ENG^high^ fibroblasts, especially cluster Fib_3, had a significant tumor-promoting function. GSVA analysis demonstrated that apoptosis signaling, reactive oxygen species signaling, oxidative phosphorylation, inflammatory response, p53 pathway, and interferon alpha signaling pathways were inhibited in ENG^high^ CAFs, while WNT/β-catenin signaling, TGFβ signaling, PI3K/AKT/mTOR signaling, G2M checkpoint, and E2F TARGET were upregulated. These results highlighted the critical role of tumor stromal cells in ccRCC development. However, there was also a significant group of tumor-suppressive fibroblasts, namely, cluster Fib_5, which were ENG^low^ cells with high expression of MHC-II molecules. GSVA scores showed that inflammatory response was upregulated in these cells, indicating that ENG expression in CAFs serve as an indicator of fibroblasts contribution to tumor growth.

Finally, the ECs in the tumor stromal cells were analyzed. Among tumor-associated ECs, cluster 0/2/3/6/8 ECs were unique to tumor samples (Figure 6A). These cells were mainly involved in angiogenesis, cell migration, protein synthesis, and extracellular matrix remodeling, implying their immunosuppressive functions. Moreover, cluster 3 and cluster 8 cells were involved in antigen presentation, which may contribute to tumor immune tolerance by enhancing antigen-specific regulatory T-cells (Benne et al., 2022; Casey et al., 2022). Cluster 8 ECs (COL1A1, COL1A2, COL3A1, PDGFRB, and ACTA2) showed an endothelium-mesenchymal transformation phenotype (Figure 6F), contributing to tumor progression and metastasis. Notably, cluster 4 ECs were scarce in tumor cells but represented an activated EC subset (HSPA1A^+^) with MHC II-restricted antigen-presenting function, suggesting potential antitumor effects. Generally, there were several subpopulations of ECs in the TME, and different subpopulations play distinct or even contradictory functions in the TME.

In conclusion, we performed bioinformatic analysis of publicly available single-cell sequencing data to elucidate the intratumoral heterogeneity in the ccRCC TME. The results of this study contribute to the understanding of the TME in human ccRCC, and provide valuable information for targeted therapy.

## Data Availability

The original contributions presented in the study are included in the article/Supplementary Material, further inquiries can be directed to the corresponding authors.

## Supplementary material

SUPPLEMENTARY FIGURE S1 scRNA-seq profiling of the landscape of ccRCC. **(A, B)** UMAP plot of single cells derived from ccRCC and para-tumor samples, with each color coded for **(A)** 30 clusters of cells, and **(B)** tissue sample type, before quality control and removal of batch effect. **(C, D)** UMAP plot of all the single cells derived from ccRCC and para-tumor samples, with each color coded for **(C)** tissue sample type, and **(D)** cluster in the cancer tissues (CT) and the para-cancer tissues (PCT), after quality control and removal of batch effect.

SUPPLEMENTARY FIGURE S2 CNV analysis for validation and expression of MALAT1 in TCGA-KIRC.** (A)** CNV analysis of each cluster of cells that originated from the scRNA-seq profiles for validation. **(B)** Expression of MALAT1 in TCGA-KIRC based on tumor grade.

SUPPLEMENTARY FIGURE S3 The CNV heterogeneity was shown in tumor cells. **(A–C)** UMAP plot of the analyzed cells, colored according to **(A)** origins (normal or tumor), **(B)** individual patient, and **(C)** cell cycle. **(D)** Metabolic pathway analysis of normal group, high CNV group, and low CNV group.

SUPPLEMENTARY FIGURE S4 Myeloid cells have a negative immunoregulatory function in ccRCC. **(A)** UMAP plot of myeloid cells derived from tumor tissues and the para-tumor tissues, respectively, colored according to cluster. **(B)** Bubble chart showing the expression level of top 10 differentially expressed genes (DEGs) of monocytes. **(C)** Violin plots of functional genes for macrophages. Tumor tissues were colored red, while normal tissues were colored green. **(D)** Violin plots of functional genes for dendritic cells. **(E)** Bubble chart showing the expression level of top 10 DEGs of dendritic cells.

SUPPLEMENTARY FIGURE S5 Correlation analysis of immune cell abundance and signaling pathways. **(A, B)** The abundance of myeloid DC cells was significantly positively correlated with the abundance of **(A)** T-cells and **(B)** cytotoxic lymphocytes. **(C, D)** The abundance of CD8+T-cells was positively correlated with **(C)** REGULATION_OF_ADAPTIVE_IMMUNE_RESPONSE and **(D)** REGULATION_OF_T_CELL_COSTIMULATION.

SUPPLEMENTARY FIGURE S6 Gene and signaling pathway expression profiles of tumor-associated fibroblasts in different clusters. **(A)** Bubble chart showing the expression level of the top 10 differentially expressed genes (DEGs) between ENGhigh CAFs (cluster fib_3 and 4) and ENGlow CAFs (cluster fib_1/2/5/6). **(B)** Differences in 50 hallmark pathway activities scored using the GSVA package, with t values calculated using a linear model.

SUPPLEMENTARY FIGURE S7 The correlation between the abundance of tumor-associated fibroblasts and signaling pathways in the TCGA-KIRC database. **(A–F)** The abundance of fibroblasts was positively correlated with (A) INFLAMMATORY_RESPONSE, **(B)** RESPONSE_TO_ROS, **(C)** APICAL_JUNCTION_ASSEMBLY, **(D)** REGULATION_OF_SPROUTING_ANGIOGENESIS, **(E)** REGULATION_OF_CELL_CYCLE_CHECKPOINT, and **(F)** REGULATION_OF_DNA_REPAIR.
