# Supplementary material for: Mapping the tumor microenvironment in clear cell renal carcinoma by single-cell transcriptome analysis
Source: Front Genet. 2023 Jul 18;14:1207233. doi: 10.3389/fgene.2023.1207233 (PMC10392130; doi:10.3389/fgene.2023.1207233)
Supplement: Supplementary file 2 [file DataSheet2.ZIP › Supplementary_Materials_Presentation/Supplementary_Materials.docx]

Supplementary Material

Mapping the tumor microenvironment in clear cell renal carcinoma by single-cell transcriptome analysis: a bioinformatics study

Yuxiong Wang^1^, Yishu Wang^2^, Bin Liu^1^, Zehua Zhang^1^, Xiaochen Su^1^, Xin Gao^1^, Yunkuo Li^1^, Faping Li^1,*^, Honglan Zhou^1,*^

^1^Department of Urology, the First Hospital of Jilin University, Changchun 130021, Jilin, China

^2^Key Laboratory of Pathobiology, Ministry of Education, Jilin University, Changchun 130021, Jilin, China

*** Correspondence:**lfping@jlu.edu.cn (F.L.);

hlzhou@jlu.edu.cn (H.Z.).

# Supplementary Figures


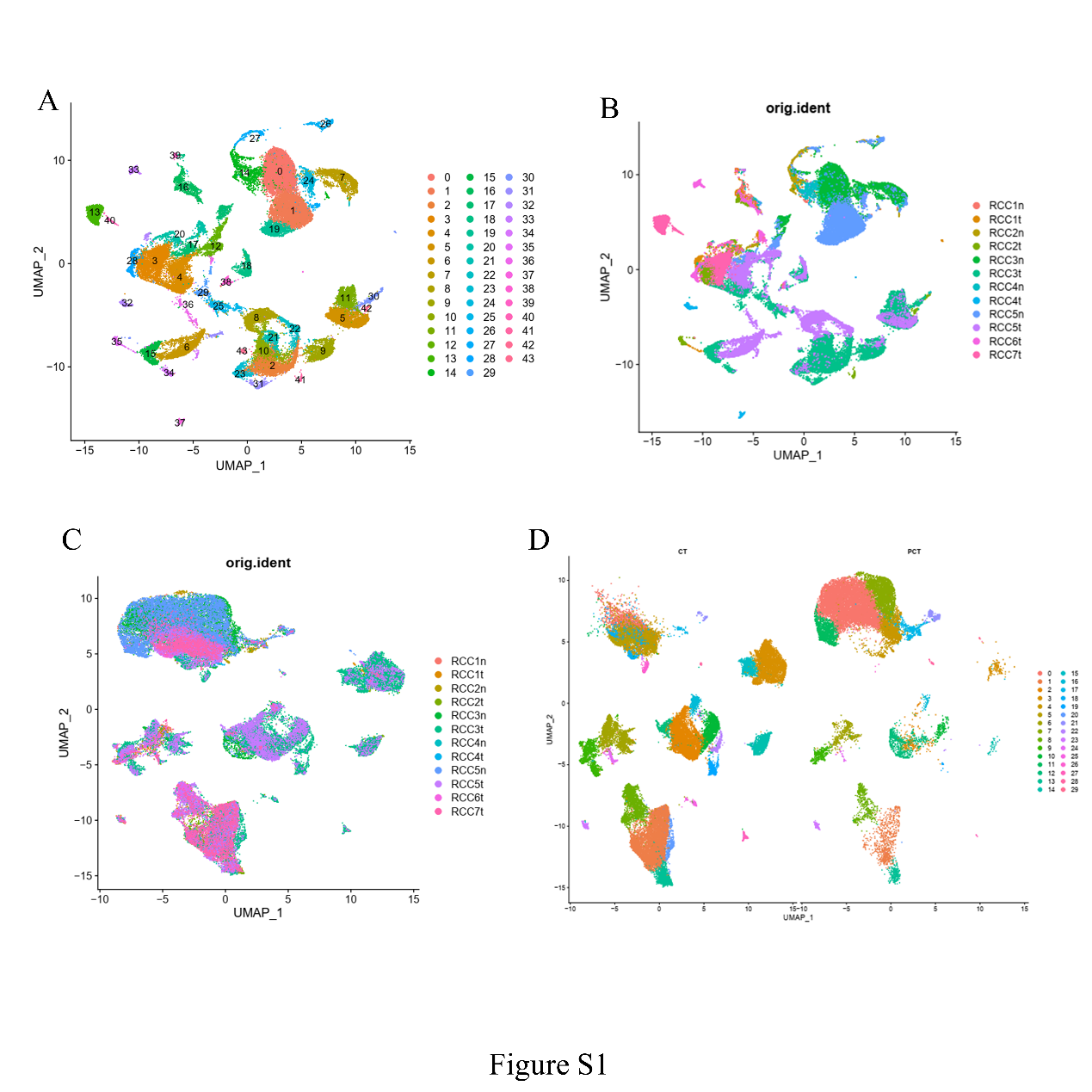


**Supplementary Fig. 1.** scRNA-seq profiling of the landscape of ccRCC. (A-B) UMAP plot of single cells derived from ccRCC and para-tumor samples, with each color coded for (A) 30 clusters of cells, and (B) tissue sample type, before quality control and removal of batch effect. (C-D) UMAP plot of all the single cells derived from ccRCC and para-tumor samples, with each color coded for (C) tissue sample type, and (D) cluster in the cancer tissues (CT) and the para-cancer tissues (PCT), after quality control and removal of batch effect.


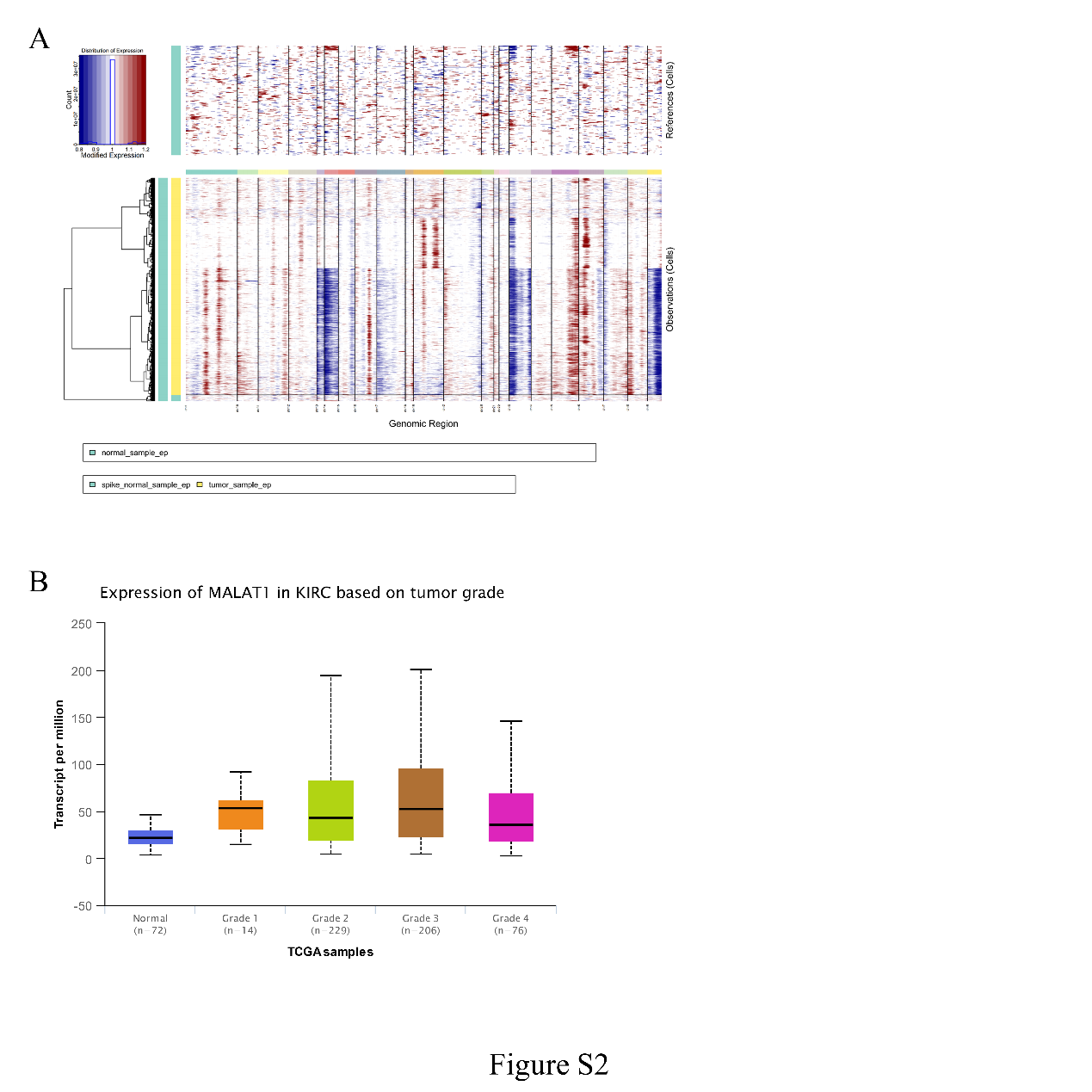


**Supplementary Fig. 2.** CNV analysis for validation and expression of MALAT1 in TCGA-KIRC. (A) CNV analysis of each cluster of cells that originated from the scRNA-seq profiles for validation. (B) Expression of MALAT1 in TCGA-KIRC based on tumor grade.


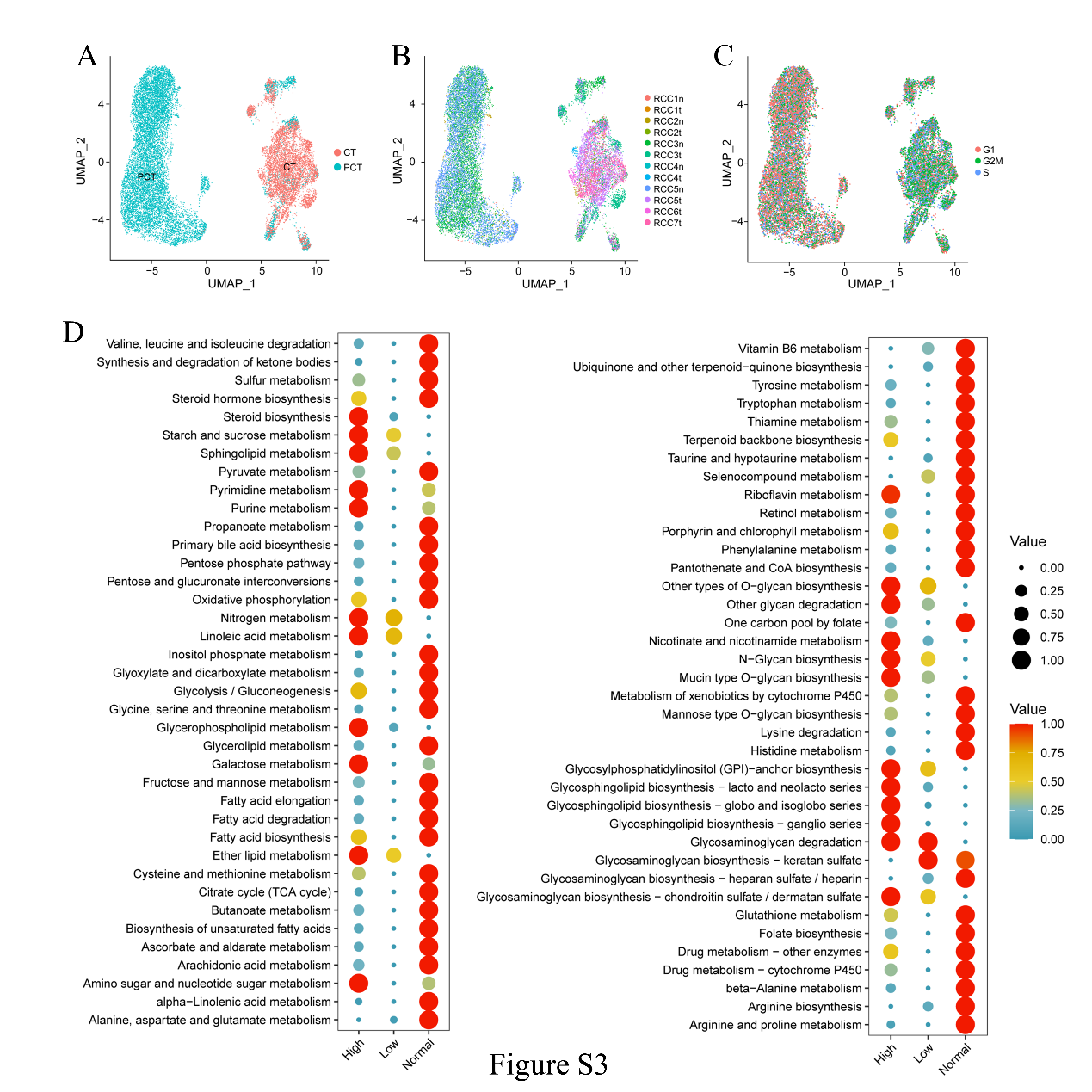


**Supplementary Fig. 3.** The CNV heterogeneity was shown in tumor cells. (A-C) UMAP plot of the analyzed cells, colored according to (A) origins (normal or tumor), (B) individual patient, and (C) cell cycle. (D) Metabolic pathway analysis of normal group, high CNV group, and low CNV group.


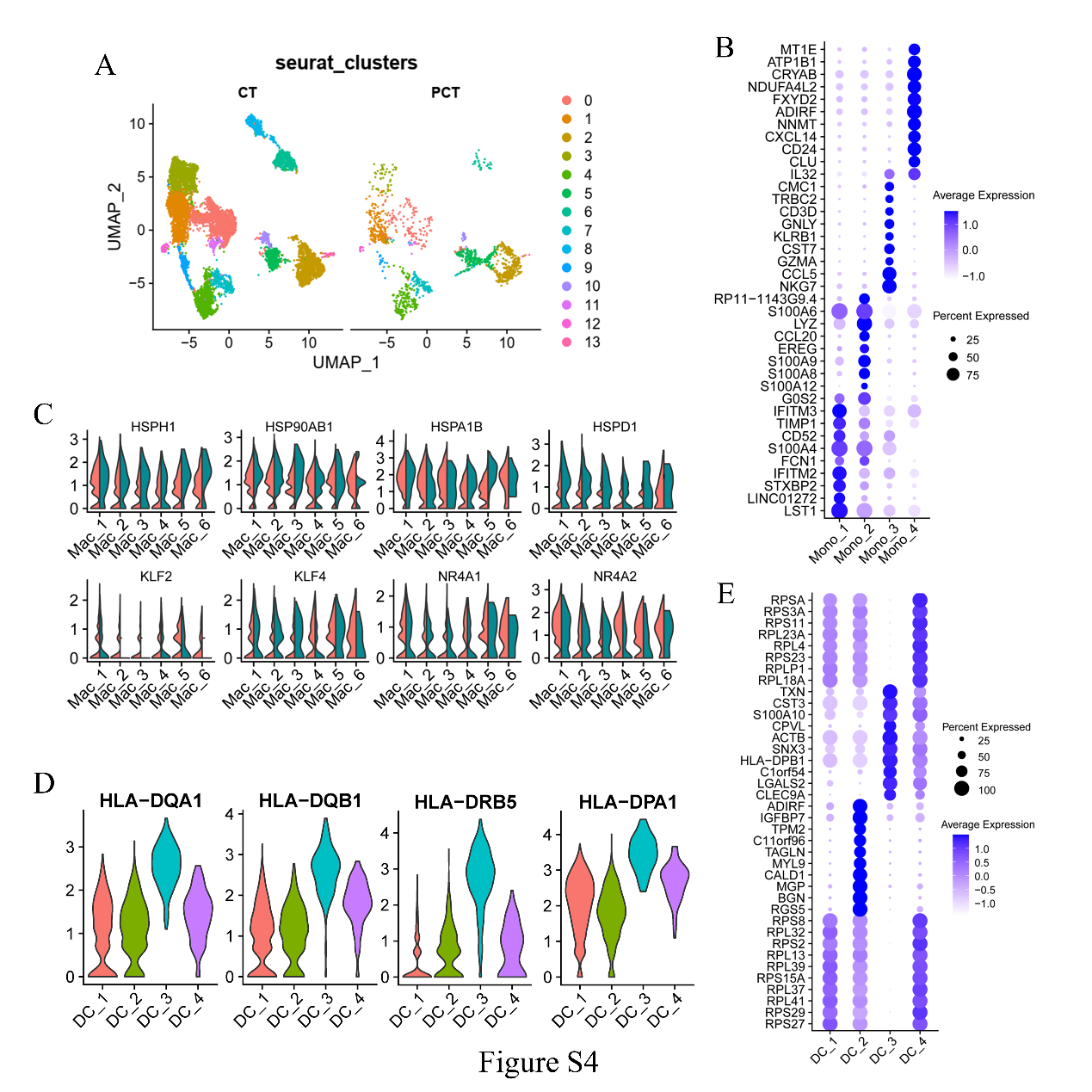


**Supplementary Fig. 4.** Myeloid cells have a negative immunoregulatory function in ccRCC. (A) UMAP plot of myeloid cells derived from tumor tissues and the para-tumor tissues, respectively, colored according to cluster. (B, E) Bubble chart showing the expression level of top 10 differentially expressed genes (DEGs) (B) monocytes and (E) dendritic cells. (C) Violin plots of functional genes for macrophages. Tumor tissues were colored red, while normal tissues were colored green. (D) Violin plots of functional genes for dendritic cells.


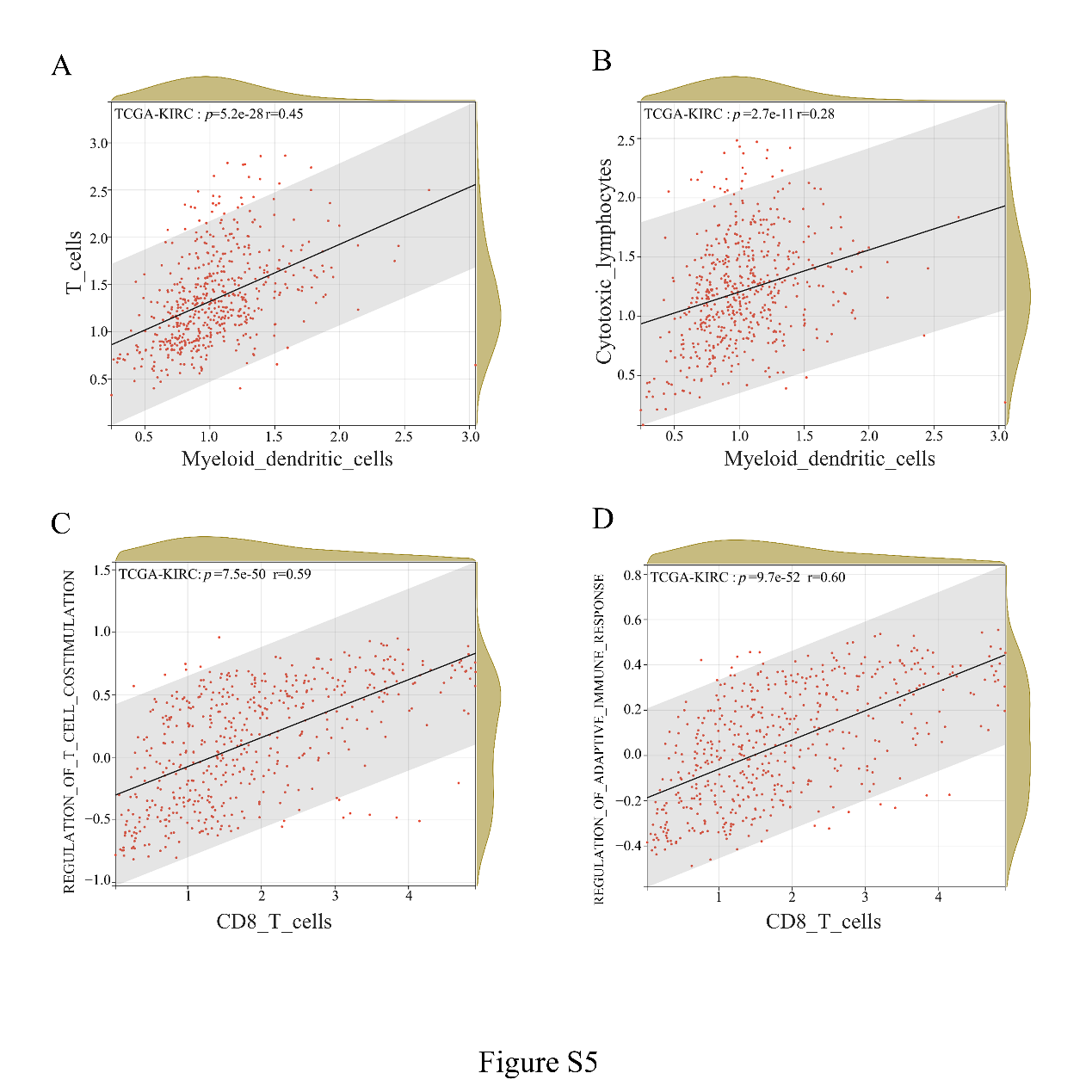


**Supplementary Fig. 5.** Correlation analysis of immune cell abundance and signaling pathways. (A-B) The abundance of myeloid DC cells was significantly positively correlated with the abundance of (A) T cells and (B) cytotoxic lymphocytes. (C-D) The abundance of CD8+T cells was positively correlated with (C) REGULATION_OF_ADAPTIVE_IMMUNE_RESPONSE and (D) REGULATION_OF_T_CELL_COSTIMULATION.


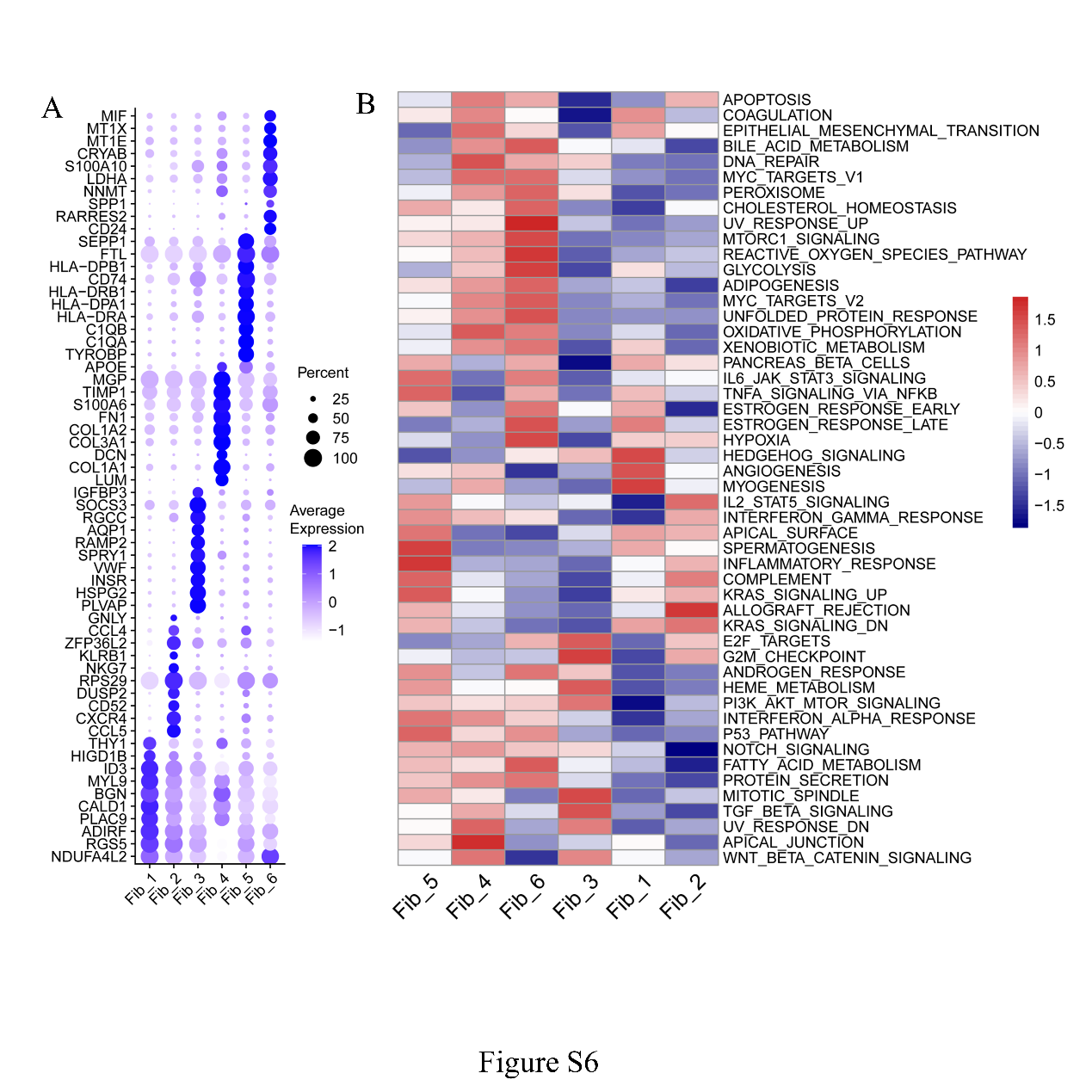


**Supplementary Fig. 6.** Gene and signaling pathway expression profiles of tumor-associated fibroblasts in different clusters. (A) Bubble chart showing the expression level of the top 10 differentially expressed genes (DEGs) between ENG^high^ CAFs (cluster fib_3 and 4) and ENG^low^ CAFs (cluster fib_1/2/5/6). (B) Differences in 50 hallmark pathway activities scored using the GSVA package, with t values calculated using a linear model.


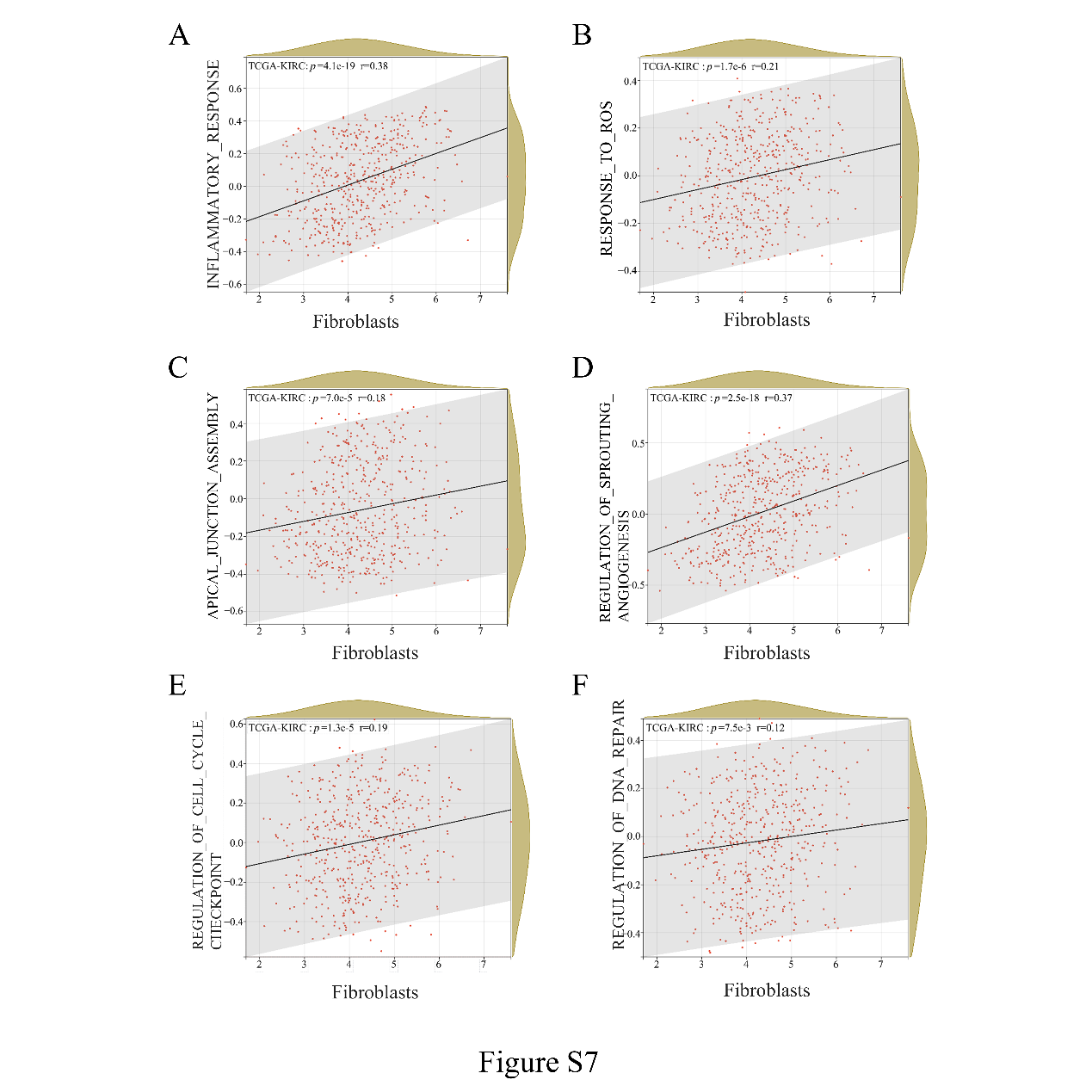


**Supplementary Fig. 7.** The correlation between the abundance of tumor-associated fibroblasts and signaling pathways in TCGA-KIRC database. (A-F) The abundance of fibroblasts was positively correlated with INFLAMMATORY_RESPONSE, RESPONSE_TO_ROS, APICAL_JUNCTION_ASSEMBLY, REGULATION_OF_SPROUTING_ANGIOGENESIS, REGULATION_OF_CELL_CYCLE_CHECKPOINT, and REGULATION_OF_DNA_REPAIR.
