# Supplementary figures and images for: Mapping the tumor microenvironment in clear cell renal carcinoma by single-cell transcriptome analysis
Source: Front Genet. 2023 Jul 18;14:1207233. doi: 10.3389/fgene.2023.1207233 (PMC10392130; doi:10.3389/fgene.2023.1207233)

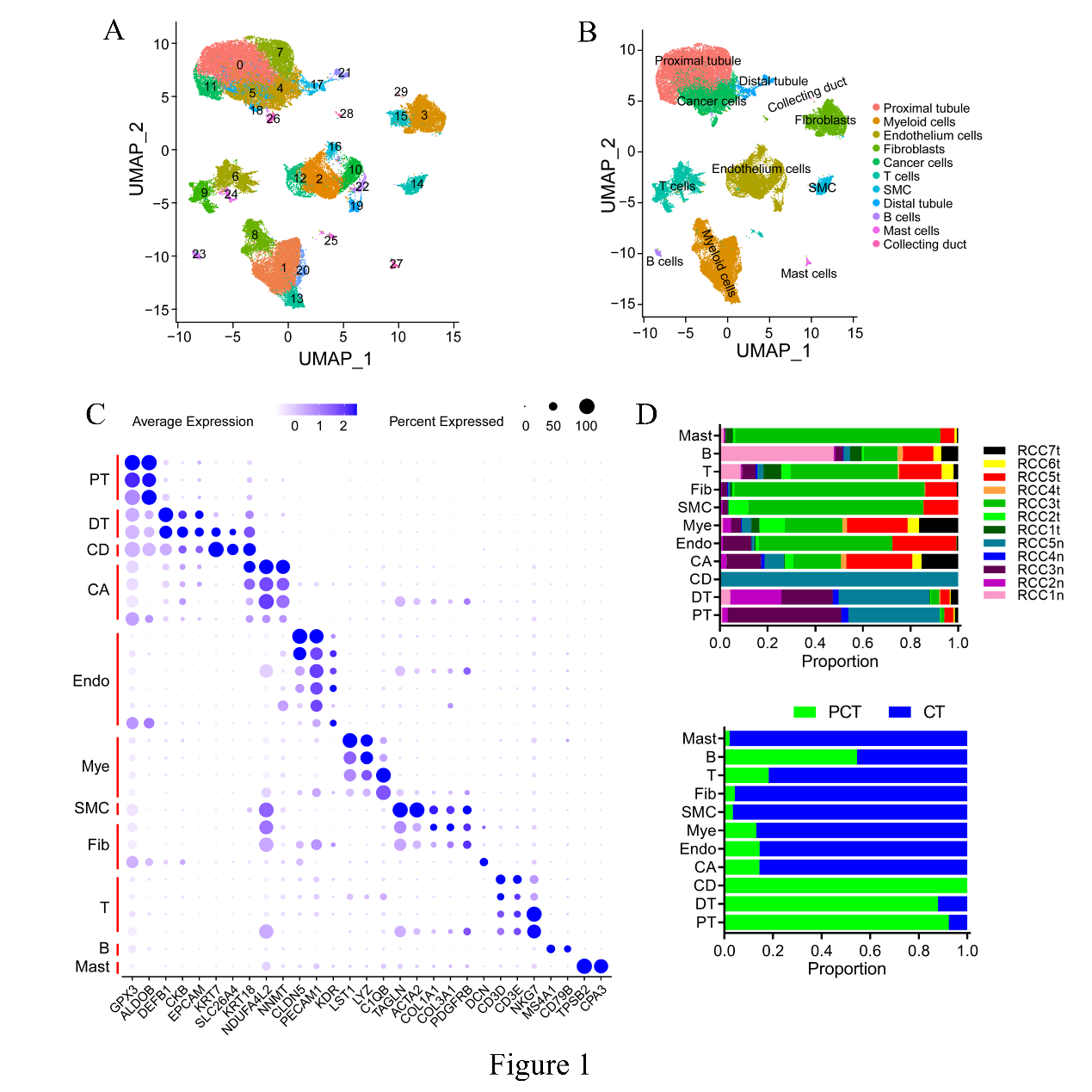

Supplement: Supplementary file 2 [file DataSheet2.ZIP › Supplementary_Materials_Presentation/Figure_1.docx]

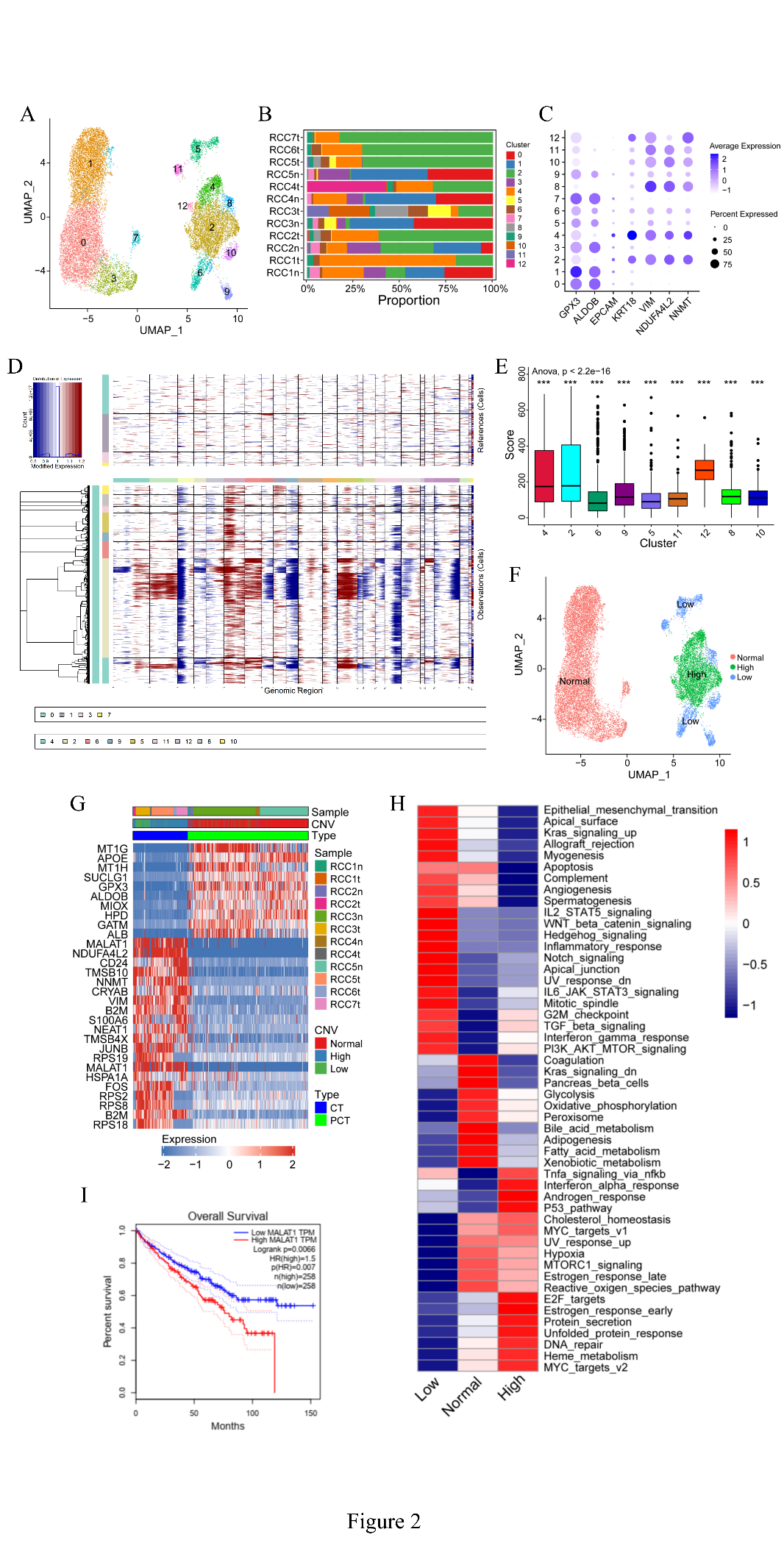

Supplement: Supplementary file 2 [file DataSheet2.ZIP › Supplementary_Materials_Presentation/Figure_2.docx]

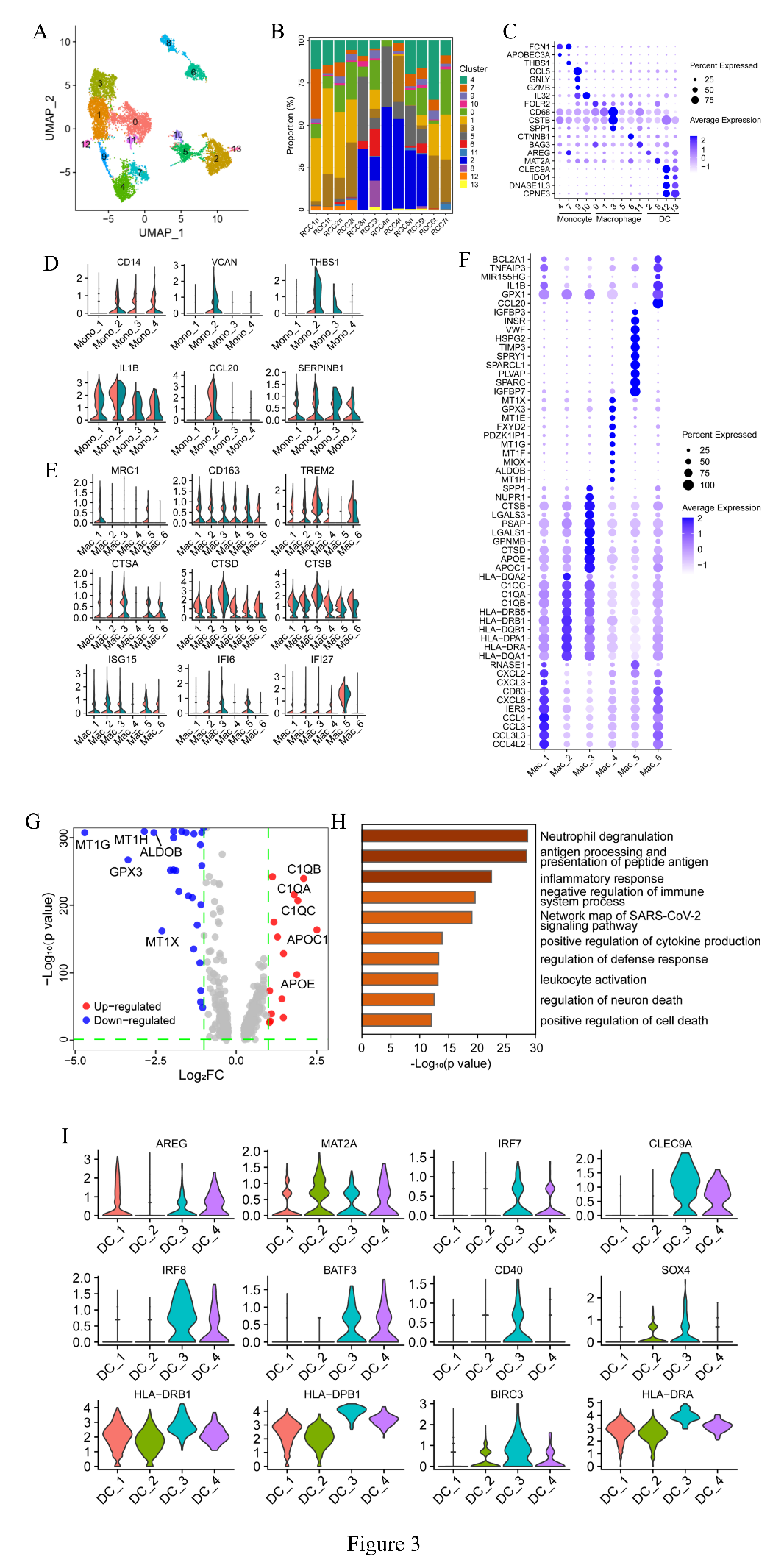

Supplement: Supplementary file 2 [file DataSheet2.ZIP › Supplementary_Materials_Presentation/Figure_3.docx]

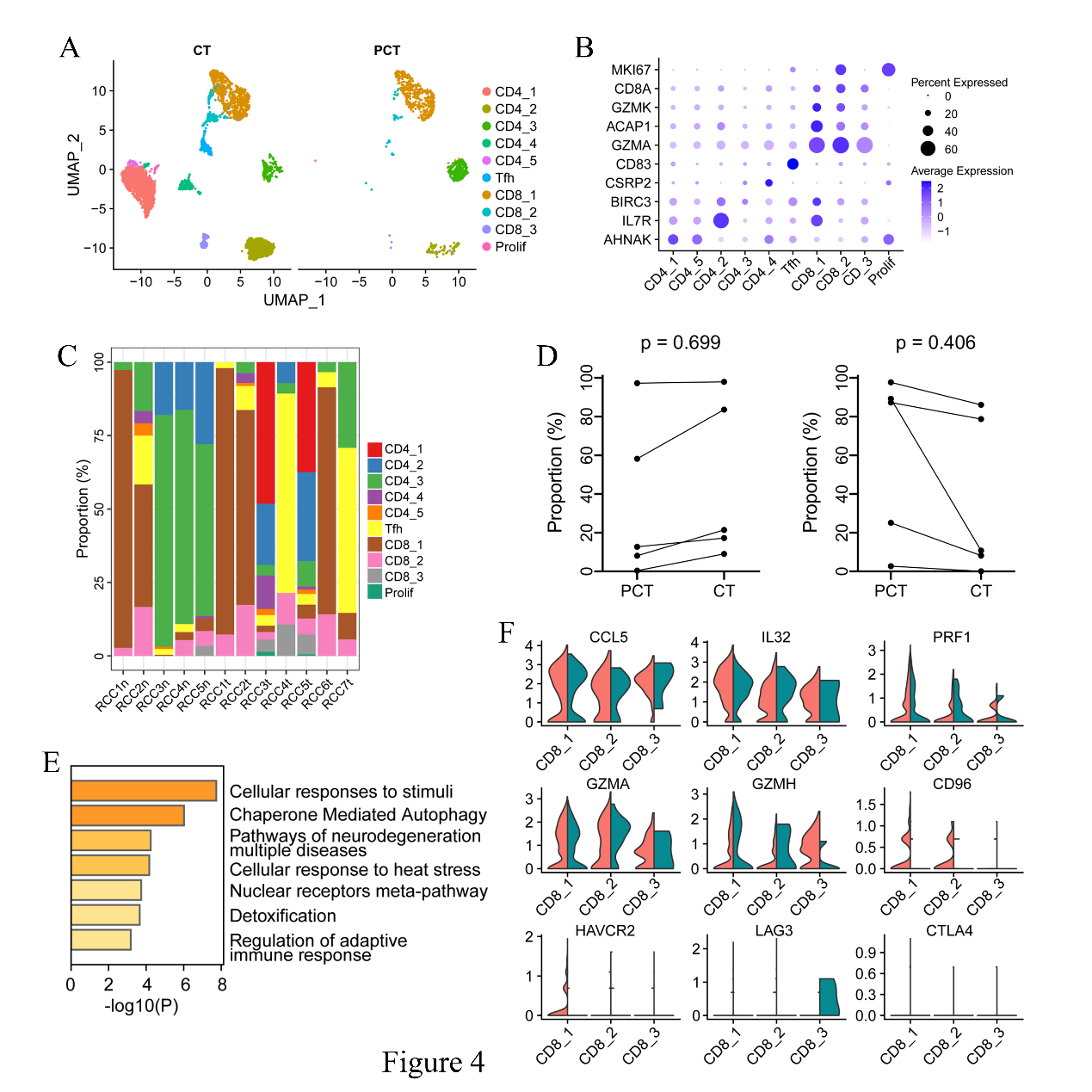

Supplement: Supplementary file 2 [file DataSheet2.ZIP › Supplementary_Materials_Presentation/Figure_4.docx]

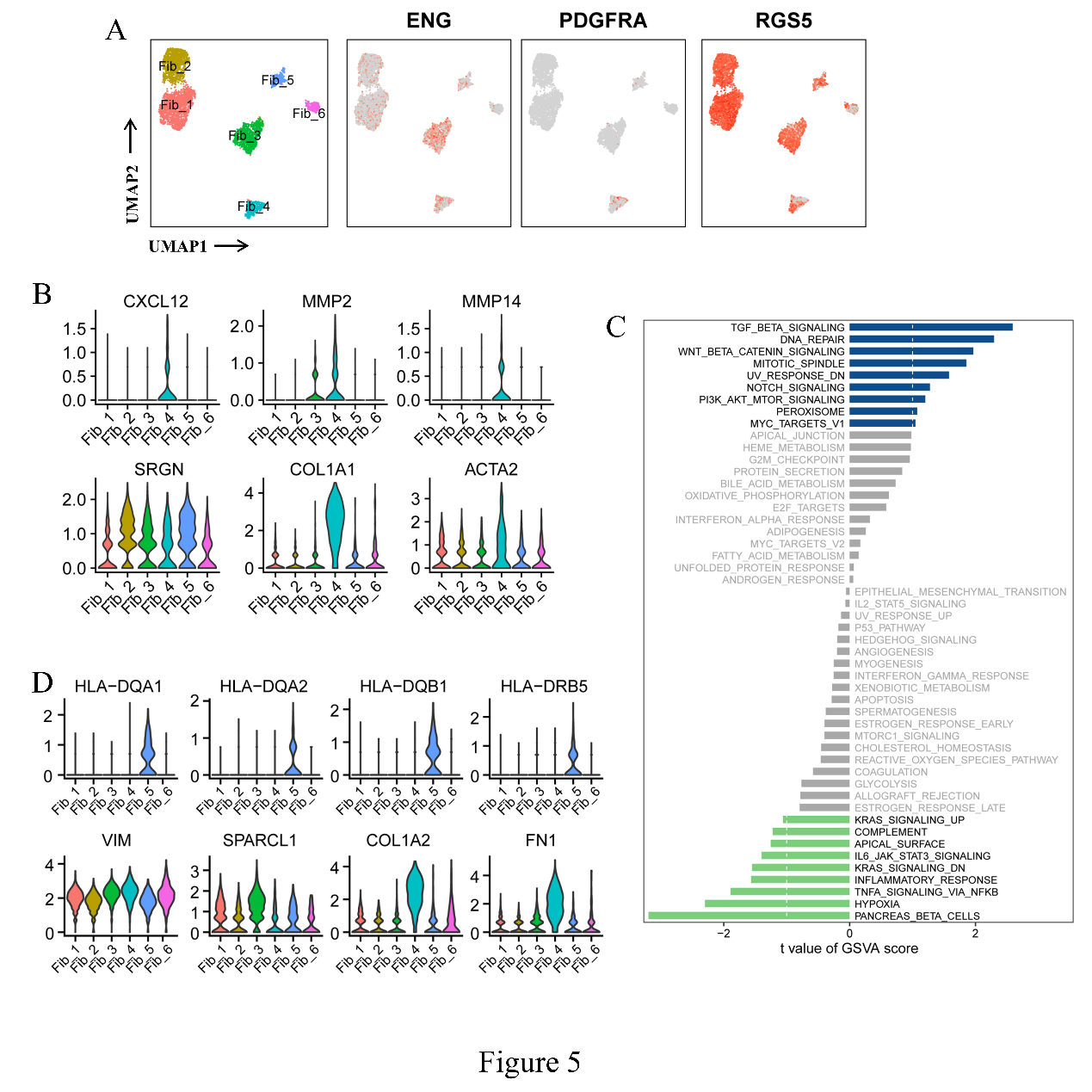

Supplement: Supplementary file 2 [file DataSheet2.ZIP › Supplementary_Materials_Presentation/Figure_5.docx]

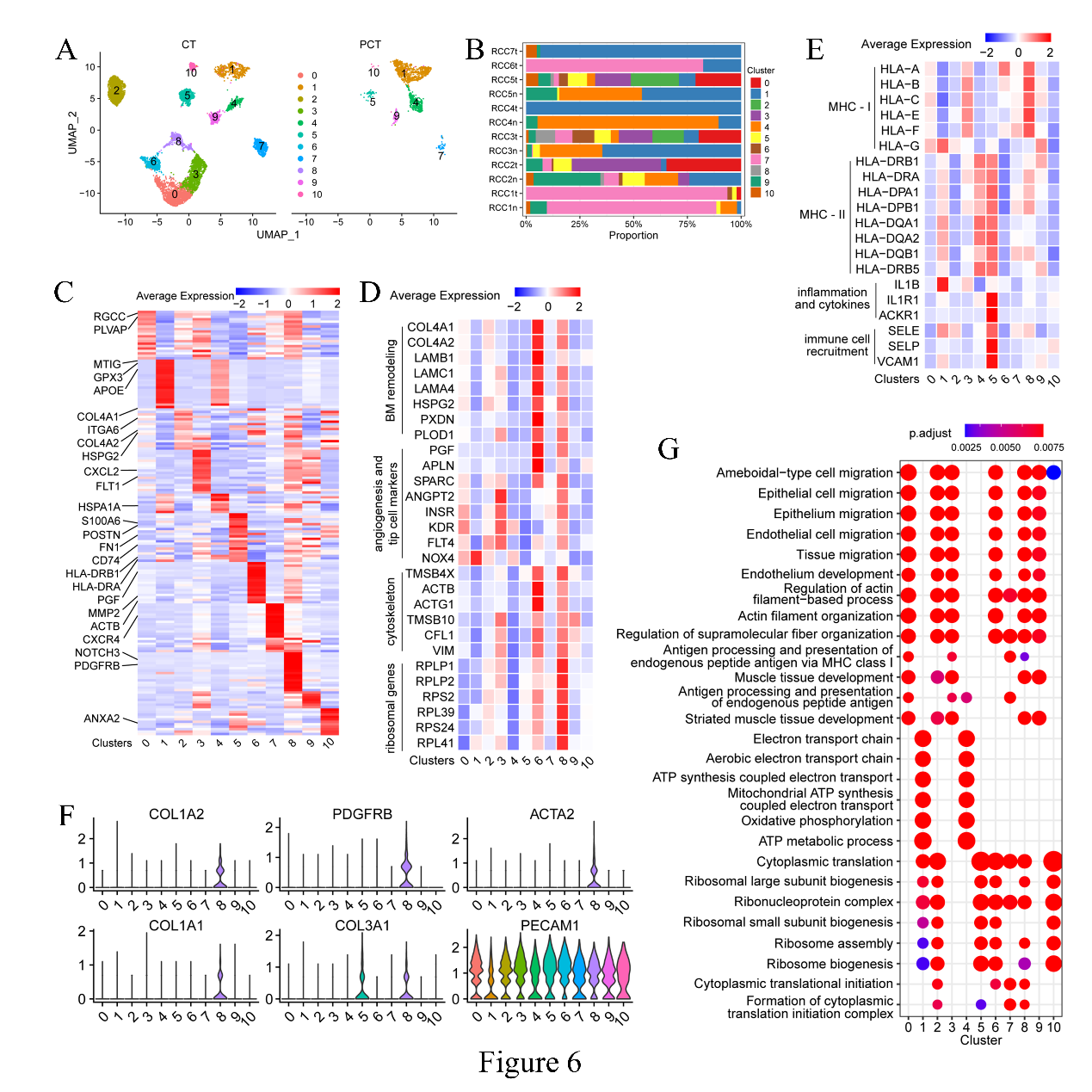

Supplement: Supplementary file 2 [file DataSheet2.ZIP › Supplementary_Materials_Presentation/Figure_6.docx]
